# Covalent Peptide‐Based N‐Myc/Aurora‐A Inhibitors Bearing Sulfonyl Fluoride Warheads

**DOI:** 10.1002/psc.70086

**Published:** 2026-02-03

**Authors:** Robert S. Dawber, Diana Gimenez, George W. Preston, Megan H. Wright, Richard Bayliss, Stuart L. Warriner, Andrew J. Wilson

**Affiliations:** ^1^ School of Chemistry University of Leeds Leeds UK; ^2^ School of Chemistry University of Birmingham Edgbaston UK; ^3^ School of Molecular and Cellular Biology University of Leeds Leeds UK; ^4^ Astbury Centre for Structural Molecular Biology University of Leeds Leeds UK

## Abstract

Orthosteric inhibition of the N‐Myc/Aurora‐A protein–protein interaction (PPI) represents a potential mechanism by which degradation of N‐Myc can be induced, given its interaction with Aurora‐A competes with the factors that tag it for proteasomal degradation. As such, this would constitute an approach for the development of drugs to treat neuroblastoma, a childhood cancer that depends upon N‐Myc. Reactive electrophiles have proven useful in the context of targeted covalent inhibitors, and in this work, we sought to improve the potency of a previously identified N‐Myc‐derived peptide by introducing a sulfonyl fluoride warhead. We successfully demonstrated selective labelling of Aurora‐A using the resultant peptidomimetics and established this labelling as recognition‐directed, providing valuable insight for further future development of N‐Myc peptidomimetics and further broadening the use of aryl sulfonyl fluoride warheads in the context of peptidomimetic PPI inhibitors.

## Graphical abstract

N‐Myc‐derived peptidomimetics bearing aryl sulfonyl fluoride warheads were shown to act as effective N‐Myc/Aurora‐A PPI inhibitors, selectively labelling Aurora‐A in a recognition‐directed manner.

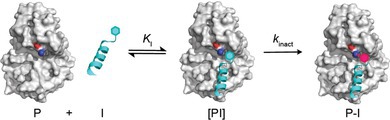

## 1 Introduction

The development of targeted covalent inhibitors (TCIs) has seen a resurgence in chemical biology and drug discovery over the past decade [1, 2, 3]. Although there are a number of TCI‐based therapeutics (e.g., penicillin), their covalent mechanism of action was often a serendipitous discovery, whilst the rational design of TCIs has historically been tainted by concerns with off‐target reactivity and associated cytotoxicity. In theory, covalency offers lucrative advantages over noncovalent inhibitors, with enhanced potency and duration of action reducing the need for high and frequent dosing [4, 5]. Recent advances in the design of suitably reactive electrophilic warheads and the expansion of the targetable proteome (and interactome) have fostered a renaissance in the discovery and development of covalent inhibitors. Development of TCIs has been dominated by cysteine‐reactive probes, with acrylamide derivatives offering a combination of desirable reactivity and biocompatibility [6]. For example, extensive efforts have incorporated acrylamide functionalities into reversible ATP‐competitive kinase inhibitors to target accessible cysteines in and around the ATP‐binding site [7]. This has given such scaffolds a new lease of life in the clinic. The development of covalent protein–protein interaction (PPI) inhibitors [8] is less well explored than covalent active site inhibitors, whilst covalent peptidomimetic inhibitors [9] are similarly less well developed. This provides broader motivation for further development of these approaches.

Aurora‐A is a mitotic kinase that has served as a potential target for anticancer drug discovery. Despite the availability of potent and selective active site kinase inhibitors [10], Aurora‐A has key roles in normal biological function [11], and none of these inhibitors have been approved for clinical use. Similarly, N‐Myc is associated with neuroblastoma, a cancer that affects children [12]. Aurora‐A interacts with N‐Myc [13] to stabilize it against degradation [13, 14]; thus, a potential strategy to treat neuroblastoma is to disrupt the N‐Myc/Aurora‐A interaction; this has been achieved allosterically with active‐site inhibitors [15, 16] and using PROTACS that degrade Aurora‐A [17]; however, orthosteric inhibition of N‐Myc/Aurora‐A might be advantageous from the perspective of not impacting the essential catalytic functions of Aurora‐A. We previously described peptide‐based competitive inhibitors of the N‐Myc/Aurora‐A interaction and found that judicious introduction of a constraint could improve the potency of the peptidomimetic relative to the unconstrained sequence [18]. Despite this, the potencies were high μM, which we considered suboptimal for further development. We hypothesized that a covalent peptidomimetic might improve the potency and offer additional advantages. Herein, we describe our efforts to develop covalent N‐Myc peptidomimetics bearing sulfonyl fluorides for Aurora‐A.

## 2 Materials and Methods

### 2.1 Peptide Syntheses and Purification

#### 2.1.1 Manual Peptide Synthesis

##### 2.1.1.1 *Method A*: Resin Swelling

The required quantity of resin was placed in a fritted empty SPE tube, DCM (3 mL) was added and the resin was agitated on a Stuart Rotator‐SB2 for 2 h to allow swelling of the resin.

##### 2.1.1.2 *Method B*: Deprotection of N‐Fmoc Protecting Groups

N‐terminal Fmoc protecting groups were removed by the addition of 20% piperidine in DMF (5 × 3 mL × 2 min), followed by rinsing the resin with DMF (5 × 3 mL × 2 min). Successful deprotection was determined by a positive colour test (*Method C*).

##### 2.1.1.3 *Method C*: Kaiser Test

The Kaiser Test was employed for the determination of the successful coupling or deprotection of N‐terminal residues. A small number of resin beads were rinsed in ethanol and placed in a vial, followed by the addition of two drops of each of the two solutions in the following order: (1) ninhydrin (5% w/v) in ethanol; (2) phenol (80% w/v) in ethanol; (3) 1 mM KCN in pyridine (2% v/v). The solution was then heated to 150°C for 1 min. A successful coupling gave no change in the colour of the beads, whereas bright blue beads illustrate the presence of a free primary amine and thus a successful deprotection.

##### 2.1.1.4 *Method D*: Coupling of Amino Acids

The desired amino acid (5 *eq*), DIPEA (5 *eq*) and coupling reagent (HCTU; 5 *eq*) were dissolved in DMF (3 mL) and added to the resin, followed by agitation for 1 h. For double couplings, this step was repeated. After removal of the reagents by filtration, the resin was washed with DMF (3 × 3 mL × 2 min), and the success of coupling was determined by a negative colour test (*Method C*). Deprotection of the Fmoc‐protected N‐terminus then followed (*Method B*).

##### 2.1.1.5 *Method E*: Coupling of FAM‐Fluorophore (via Ahx Linker)

6‐(Fmoc‐amino)hexanoic acid (5 *eq*), DIPEA (5 *eq*) and coupling reagent (HCTU; 5 *eq*) were dissolved in DMF (3 mL) and added to the resin, followed by agitation for 1 h. After removal of the reagents by filtration, the resin was washed with DMF (3 × 3 mL × 2 min), and the success of coupling was determined by a negative colour test (*Method C*). Deprotection of the Fmoc‐protected N‐terminus then followed (*Method B*). 5,6‐carboxyfluorescein (5 *eq*), DIPEA (5 *eq*) and HCTU (5 *eq*) were dissolved in DMF (3 mL) and added to the resin, followed by agitation for 1 h (shielded from light). After removal of the reagents by filtration, the resin was washed with DMF (3 × 3 mL × 2 min) ahead of cleavage and deprotection (*Method G*).

##### 2.1.1.6 *Method F*: N‐Terminal Acetylation

Acetic anhydride (10 *eq*) and DIPEA (10 *eq*) were dissolved in DMF (3 mL), and the solution was transferred to the resin. After 2 h, the resin was drained, washed with DMF (3 × 2 mL × 2 min), and successful capping was determined by a negative colour test (*Method C*).

##### 2.1.1.7 *Method G*: Cleavage and Deprotection of Rink Amide MBHA Resin

After peptide synthesis and N‐terminal capping were complete, the resin was washed with DMF (5 × 3 mL × 2 min), DCM (5 × 3 mL × 2 min) and then Et_2_O (3 × 3 mL × 2 min). Peptides were then simultaneously cleaved and side‐chain deprotected with a prepared Reagent K cleavage cocktail (3 mL): TFA/phenol/H_2_O/thioanisole/EDT (82.5/5/5/5/2.5). After 3 h, the resin was washed with fresh TFA (3 mL × 2 min), and the solution was concentrated under a flow of nitrogen. The resulting oil was precipitated with ice‐cold Et_2_O (10 mL) and placed in a centrifuge (3000 rpm × 3 min). The supernatants were removed, the precipitate rinsed with ice‐cold Et_2_O (3 × 10 mL) and dried under a flow of nitrogen.

##### 2.1.1.8 *Method H*: On‐Resin Ethene Sulfonyl Fluoride (ESF) Incorporation

ESF (5 *eq*) was dissolved in DMF (3 mL) and added to the resin, followed by agitation for 3 h. Reagents were removed by filtration and the resin was washed with DMF (3 × 3 mL × 2 min). The process was then repeated once more.

##### 2.1.1.9 *Method I*: On‐Resin Aryl Sulfonyl Fluoride (ASF) Incorporation

3‐(Fluorosulfonyl)benzoic acid (5 *eq*), DIPEA (5 *eq*) and coupling reagent (HCTU; 5 *eq*) were dissolved in DMF (3 mL) and added to the resin, followed by agitation for 3 h. Reagents were removed by filtration, and the resin was washed with DMF (3 × 3 mL × 2 min). The process was then repeated once more.

#### 2.1.2 Cycles for Automated Microwave‐Assisted Peptide Synthesis

Peptides that were synthesized by automated microwave‐assisted Fmoc‐SPPS followed this cycle on the peptide synthesizer (CEM Liberty Blue):

##### 2.1.2.1 Resin Loading

Clean reaction vessel; wash with DMF; wash with DCM; transfer resin to reaction vessel; wash with DMF; wash with DCM; transfer resin to reaction vessel; wash with DMF; wash with DCM; vessel draining.

##### 2.1.2.2 Deprotection

Clean resin dip tube, wash with DMF (15 mL), add DMF:piperidine:formic acid (75:20:5) solution (6 mL), microwave method (30 s), wash with DMF (15 mL), add DMF:piperidine:formic acid (75:20:5) solution (6 mL), microwave method (30 s), wash with DMF (15 mL), clean resin dip tube, wash with DMF (15 mL).

##### 2.1.2.3 Coupling

Add amino acid solution (0.2 M, 2.5 mL), add coupling reagent (DIC; 0.2 M, 1 mL), add activator base (oxyma; 0.2 M, 0.5 mL), microwave method (3 min), wash with DMF (15 mL), drain. For double couplings, this step was repeated.

After the final residue, the resin was ejected from the reaction vessel, and N‐terminal acetylation/modification (*Methods E*, *F*, *G*, *K* or *L*) and cleavage and deprotection (*Method H*) were performed manually.

#### 2.1.3 Cycles for Automated Room Temperature Peptide Synthesis

Peptides that were synthesized by automated room temperature Fmoc‐SPPS followed this cycle on the peptide synthesizer (CEM Liberty Blue):

##### 2.1.3.1 Resin Loading

Clean reaction vessel; wash with DMF; wash with DCM; transfer resin to reaction vessel; wash with DMF; wash with DCM; transfer resin to reaction vessel; wash with DMF; wash with DCM; vessel draining.

##### 2.1.3.2 Deprotection

Clean resin dip tube, wash with DMF (15 mL), add DMF:piperidine:formic acid (75:20:5) solution (6 mL), room temperature method (5 min), wash with DMF (15 mL), add DMF:piperidine:formic acid (75:20:5) solution (6 mL), room temperature method (5 min), wash with DMF (15 mL), clean resin dip tube, wash with DMF (15 mL).

##### 2.1.3.3 Coupling

Add amino acid solution (0.2 M, 2.5 mL), add coupling reagent (HCTU; 0.2 M, 1 mL), add activator base (DIPEA; 0.2 M, 0.5 mL), room temperature method (12 min), wash with DMF (15 mL), drain. For double couplings, this step was repeated.

After the final residue, the resin was ejected from the reaction vessel, and N‐terminal acetylation/modification (*Methods E, F, G, K* or *L*) and cleavage and deprotection (*Method H*) were performed manually.

#### 2.1.4 Peptide Purification

Peptides were purified by preparative scale HPLC using a Kinetex 5‐μM EVO C18 preparative column (reversed phase) on an increasing gradient of acetonitrile in water (plus 0.1% TFA v/v in water) at a flow rate of 10 mL min^−1^. Unless otherwise stated, crude peptides were dissolved in minimal amounts of either dimethyl sulfoxide or acetonitrile:water (1:1) depending on the solubility of the sequence. Purification runs injected a maximum of 5 mL of crude peptide solution and were allowed to run for 35 min, with acetonitrile increasing from 5% to 95%, and the eluent scanned with a diode array at 210, 254 and 280 nm. Fractions were checked by liquid chromatography mass spectrometry (LC–MS), concentrated in vacuo and lyophilized. Final purity of peptides was confirmed by HRMS and analytical HPLC.

#### 2.1.5 Biophysical and Biochemical Experiments

##### 2.1.5.1 Optimized Direct Binding FA Assay (Aurora‐A Protein)

Aurora‐A_119‐403, C290A/C393A_ (herein referred to as Aurora‐A, 10 µL 488 µM), or covalently modified Aurora‐A 500 μM stock solution in buffer (25‐mM Tris, 150‐mM NaCl, 5‐mM MgCl_2_ and pH 7.5) was added to the first column of wells of a Corning 384‐well microplate (low volume, black and round bottom). To all remaining wells, 5 μL of the same buffer solution was added. The first column of wells containing protein was subject to a 50% series dilution across the plate from left to right, affording a protein concentration gradient starting at 488 or 500 μM at a total volume of 5 μL per well. Fluorescently labelled peptide in the same buffer (5 μL of 100 nM stock) was added to ‘measurement’ wells and buffer alone (5 μL) to ‘control’ wells to afford a protein concentration gradient starting at 244 or 250 μM and a constant 50‐nM final concentration of the fluorescently labeled peptide in a total volume of 10 μL per well. Plates were left to equilibrate for a period of 2 h before fluorescence anisotropy (FA) was measured using an EnVision 2013 MultiLabel plate reader (Perkin Elmer) at excitation and emission wavelengths of 480 and 535 nm, respectively (dichroic mirror: 505 nm). Data points represent the mean of three measurements and control rows per peptide; error bars indicate SD. Anisotopies were determined according to Equations (1) and (2). The dependence of FA upon Aurora‐A was fitted to a one‐site total binding model by nonlinear regression according to Equations (3) and (4). Results are reported as K_d_ ± SD.

(1) $$I=\left(2\mathrm{PG}\right)+S$$

(2) $$r=\left(S-\mathrm{PG}\right)/I$$

(3) $$L_{b}=\left(r-r_{\min}\right)/\left[\lambda \left(r_{\max}-r\right)+\left(r-r_{\min}\right)\right]$$

(4) $$y=\left\{\left(k+x+\left[\mathrm{FL}\right]\right)-\sqrt{\left\{k+x+{\left[\mathrm{FL}\right]}^{2}-4^{*}\left[\mathrm{FL}\right]\right\}}\right\}/2$$

where *I* = total intensity, *r* = anisotropy, *P* = perpendicular intensity, *S* = parallel intensity, *G* is an instrument gain factor, *L*_*b*_ = fraction ligand bound, *λ* = I_bound_/I_unbound_ = 1, [*FL*] = concentration of fluorescent ligand, *k* = K_D_ and *x* = [added titrant].

##### 2.1.5.2 Optimized Competition FA Assay (Aurora A/N‐Myc)

Competitor (unlabelled peptide) solution (11 µL, 2.5 mM) in buffer (25 mM Tris, 150 mM NaCl, 5 mM MgCl_2_ and pH 7.5) was added to the first column (six rows per competitor) of wells of a Corning 384‐well microplate (low volume, black and round bottom). To all other wells in these rows (i.e., discounting the first column containing competitor), 5.5 μL of the same buffer was added. The competitor was subject to a 50% dilution series, affording a concentration gradient of competitor starting at 2.5 mM with a total volume of 5.5 μL in each well. To all wells, 5.5 μL of 45 μM Aurora‐A solution was added, with mixing, leaving a total volume of 11 μL per well. To all wells in the top three rows (i.e., ‘measurement’ wells), 5.5 μL of 150 nM N‐Myc_61–89_ FAM solution was added with mixing, resulting in a total volume of 16.5 μL per well. Thus, the final composition of these wells contained well‐mixed 16.5‐μL solutions of 15 μM Aurora‐A, 50 nM N‐Myc_61–89_ FAM and a concentration gradient of competitor peptide starting at 833 μM in the first column. To all wells in the bottom three rows (i.e., ‘control’ wells), 5.5 μL of buffer was added with mixing, resulting in wells matching the final composition above but in the absence of 50 nM N‐Myc_61–89_ FAM. FA was measured using an EnVision 2013 MultiLabel plate reader (Perkin Elmer) at excitation and emission wavelengths of 480 and 535 nm, respectively (dichroic mirror: 505 nm). Data points represent the mean of three measurement rows and three control rows per competitor; error bars indicate SD. Anisotopies were determined as before. The dependence of FA upon [competitor] was fitted to a sigmoidal (logistic) curve model (Equation 5). Results are reported as IC_50_ ± SD.

(5) $$y=r_{\max}+\left(r_{\min}-r_{\max}\right)/\left(1+{\left(x/x_{0}\right)}^{p}\right)$$

where *y* = *r* = anisotropy, and *x*_0_ = mid‐point of the curve between the *r*_*max*_ and *r*_*min*_ plateau.

#### 2.1.6 Mass‐Spectrometry Analysis of Covalent Peptides

##### 2.1.6.1 General Points

HRMS spectra were recorded on a Bruker Daltonics microTOF using electrospray ionization (ESI). Samples were injected onto a Phenomenex Aeris 3.6‐μM WIDEPORE C4 200‐Å LC column (50 × 2.1 mm) and subjected to a 3.5‐min gradient of acetonitrile +0.1% formic acid (B) in water +0.1% formic acid (A) as follows: t = 0.00–0.30 min, A = 99%–99%, B = 1%–1%, t = 0.30–0.70 min, A = 99%–97%, B = 1%–3%, t = 0.70–2.40 min, A = 97%–5%, B = 3%–95%, t = 2.40–3.00 min, A = 5%–5%, B = 95%–95%, t = 3.00–3.50 min, A = 5%–99%, B = 95%–1%.

##### 2.1.6.2 HRMS Sample Preparation for Initial Analysis

For each protein, a stock solution was diluted to 45 μM in buffer (25 mM TRIS, 150 mM NaCl, 5 mM MgCl_2_ and pH 7.5) at a total volume of 50 μL. A 10‐mM stock solution (in DMSO) of N‐Myc_73–89_ ASF was diluted to 0.5 mM in buffer (same as above) before being added to the protein sample with mixing to afford a peptide concentration of 90 μM.

##### 2.1.6.3 HRMS Sample Preparation for Kinetic Analysis

For each sample, an 800 μM stock solution of Aurora‐A_119–403 C290A/C393A_ (herein referred to as Aurora‐A) was diluted to 10 μM in buffer (25 mM TRIS, 150 mM NaCl, 5 mM MgCl_2_ and pH 7.5) at a total volume of 150 μL. A 10 mM stock solution (in DMSO) of each covalent N‐Myc peptide was diluted to 0.5 mM in buffer (same as above) before being added to the protein sample with mixing to afford the desired protein:peptide stoichiometry.

All injections were of a 3‐μL volume, and each sample was injected at 15‐min time intervals for a total period of 4 h. Aurora‐A was typically observed at a retention time of 1.95–2.20 min. All spectra were set to automatically deconvolute between 1.95 and 2.15 min and to automatically calculate the areas under peaks for unmodified protein (P), protein + inhibitor (P‐I), protein + 2 × inhibitor (I‐P‐I), protein + 3 × inhibitor (I_2_‐P‐I) and protein + 4 × inhibitor (I_2_‐P‐I_2_). The automatically calculated areas were converted to ratios in Microsoft Excel and plotted as change in total occupancy of all protein states over time.

#### 2.1.7 Protein Digests and Attempted Sequencing

##### 2.1.7.1 Protein Samples

The protein used for the experiment corresponded to residues 119–403 of full‐length human Aurora‐A with substitutions K119G, N120A, E121M, C290A and C393A. Briefly, a stock solution of Aurora‐A (488 μM and 25 μL) was diluted to a final concentration of 10 μM in 25 mM Tris, 150 ‐mM NaCl, 5 mM MgCl_2_ and pH 7.5. To this solution, 1.2‐mol equivalents of a peptide stock solution [10 mM] in DMSO were added as a single aliquot (clean DMSO only for the control unlabelled protein), and the mixtures were left to react on a rotary shaker overnight. Each reaction mixture was then cleaned from any unreacted residual probe, including the control protein with no probe, by repeated cycles of ultracentrifugation using a 10‐kDa molecular‐weight cut‐off filter (Amicon, Millipore) (5% × 5% DMSO in 25 mM Tris, 150 mM NaCl, 5 mM MgCl_2_ and pH 7.5) + (5% × 1% DMSO in 25 mM Tris, 150 mM NaCl, 5 mM MgCl_2_ and pH 7.5) + (5 × 25 mM Tris, 150 mM NaCl, 5 mM MgCl_2_ and pH 7.5). After the final wash, the sample was concentrated to a final volume of ~50 μL, and the concentration of the final protein solution was evaluated using protein Abs at λ = 280 nM in a nanodrop.

Sample aliquots containing 60 μg of the labelled and unlabelled proteins were then treated with acetone to fully precipitate the proteins (four volumes of acetone per volume of sample, −20°C, overnight), and the precipitate was pelleted by centrifugation (13,300 × rpm, 10 min). Supernatant was removed by careful aspiration with a micropipette and air‐dried.

Pellets were then reconstituted in 75 μL of Trypsin/Lys‐C Kit Rapid Digestion buffer (VA1061, Promega). Vigorous agitation was required to effect dissolution of the pellets.

##### 2.1.7.2 Protein Reduction‐Denaturation and Derivatization

To a single 75‐μL aliquot of reconstituted protein (60 μg) was added 10 μL of 1,4‐dithiothreitol (0.7 mg mL^−1^ solution in Rapid Digestion buffer), with vortex‐mixing. The mixture was incubated at 40°C for 30 min with 400 rpm shaking (Benchmark MultiTherm shaker), then allowed to cool to ambient temperature. The reduced proteins were derivatized by adding 10 μL of iodoacetamide (3.3 mg mL^−1^ solution in the same buffer), with vortex‐mixing. The mixture was left to stand at ambient temperature, in the dark, for 30 min with 400 rpm shaking. Cold acetone (380 μL, ≤ 0°C) was added, and the mixture was left to stand at −20°C overnight. The resulting suspension was centrifuged (13,300 × rpm, 10 min), and the supernatant was removed by careful aspiration with a micropipette. The pellet was washed twice with 240 μL of 4:1 acetone–water, followed by standing, centrifugation and aspiration of the supernatant. The washed pellets were allowed to air‐dry for 30 min and reconstituted in 36 μL of rapid digestion buffer. Aliquots (18 µL) from each sample was withdrawn into low‐volume HPLC vials, mixed with 4 μL of a 3% (v/v) aqueous solution of formic acid, incubated for 10 min at room temperature and then further diluted by the addition of +4 μL of H_2_O:acetonitrile 50:50% (v/v). HR‐LC/MS analysis of these samples confirmed complete derivatization of the proteins prior to digestion.

##### 2.1.7.3 Protein Trypsin Digestion

Following manufacturer instructions, 100 μg of Rapid Trypsin/Lys‐C Mix (MS Grade, Promega) was reconstituted into 100 μg of the provided resuspension buffer to make a protease concentration of 1 mg ml^−1^. This solution (4 µL) was added to each of the samples containing the remaining volume of the derivatized proteins (18 μL, ~30 μg of protein) in rapid digestion buffer. The mixtures were then briefly vortex‐mixed and incubated at 37°C overnight, with 400 rpm shaking (Benchmark MultiTherm shaker). Samples were then centrifuged briefly to bring down condensation from inside the lids of the tubes, and incubation was continued for a further 15 min. When the incubation was complete, the mixture was centrifuged briefly, and 4 μL of a 3% (v/v) aqueous solution of formic acid was added, with vortex‐mixing. The mixture was incubated for a further 10 min at 37°C with 400 rpm shaking. The whole digests were stored at −20°C and further diluted to 0.2 μg μL^−1^ for direct analysis using H_2_O/ACN/FA 45:55:0.1% (v/v) solution, or alternatively stored as dry protein pellets after three repeated cycles of precipitation followed by standing, centrifugation and aspiration of the supernatant using 100 μL of 4:1 acetone–water.

##### 2.1.7.4 Peptide Solubilization

To each digest (21 μL, ~1.15) was added 88 μL of 0.1% (v/v) formic acid in 7:3 water–acetonitrile, with vortex‐mixing. Samples were incubated at 25°C for 10 min with 800 rpm shaking (Benchmark MultiTherm Shaker). Twenty microlitres of sample was then added to 530 μL of 0.1% (v/v) aqueous formic acid. The diluted samples were incubated at 25°C for 10 min with 800 rpm shaking (Benchmark MultiTherm Shaker), then centrifuged (10,000 × *g*, 2 min) to bring down any particles. Fifty microlitres of supernatant was transferred to a polypropylene autosampler vial (‘inner cone’ style) with silicone/PFTE‐lined cap (Fisher).

##### 2.1.7.5 Reversed‐Phase Liquid Chromatography and Mass Spectrometry

Solubilized/diluted samples were analysed using a Bruker nanoElute liquid chromatograph coupled to a Bruker timsTOF Pro 2 mass spectrometer via a CaptiveSpray ion source (Bruker Daltonics, Bremen, Germany). The system was controlled using Bruker Compass HyStar 6.0 and Bruker timsControl 3.1. Samples (1 μL) were first trapped on a Thermo Scientific PepMap Acclaim C18 trap cartridge (length × i.d. = 5 mm × 300 μm, particle size = 5 μm), and then separated on an IonOpticks AURORA series Gen2 analytical column with a built‐in emitter tip (length × i.d. = 25 cm × 75 μm, stationary phase = C18, particle size = 1.6 μm, pore size = 120 Å, emitter tip i.d. = 5 μm). The analytical column was maintained at a temperature of 40°C using a Bruker Column Toaster. Eluents A and B were 0.1% (v/v) solutions of formic acid in water and acetonitrile, respectively. Gradient elution was performed at a flow rate of 0.4 μL min^−1^, with the proportion of eluent B varied as follows: linear increase of 2% to 60% over 60.0 min; then linear increase of 60% to 95% over 0.5 min; then hold at 95% for 5.6 min. The system was re‐equilibrated at the end of each run, and an extended wash was performed [wash solution 1 was 0.1% (v/v) aqueous formic acid; wash solution 2 was 0.1% (v/v) formic acid in a 1:1:1:1 mixture of acetonitrile, isopropanol, methanol and water].

The mass spectrometer was operated in positive polarity mode. The capillary voltage was 1500 V, the dry gas flow rate was 3 L min^−1^ and the temperature was 180°C. Data were acquired using the following settings: MS scan range, *m*/*z* 100–1700; scan mode, PASEF [19]; 1/K_0_ start, 0.6 V s cm^−2^; 1/K_0_ end, 1.6 V s cm^−2^; ramp time, 100 ms; accumulation time, 100 ms; ramp rate, 9.52 Hz; number of PASEF ramps per cycle, 10. Isolation width was *m*/*z*‐dependent according to Table 1. The collision energy was ion‐mobility‐dependent according to Equation (6), where *CE* is the collision energy in eV, and *K*_0_ is the reduced ion mobility in cm^−2^ V^−1^ s^−1^.

**TABLE 1:** Dependence of isolation width on *m*/*z*.

*m*/*z*	Isolation width (*m*/*z* units)
< 700	2
700–800	0.01 × *m*/*z* − 5
> 800	3

(6) $$\mathrm{CE}=\frac{1}{K_{0}}\times 39-3.4$$

##### 2.1.7.6 Computational Searches

The process for peptide identification is described below for N‐Myc_73–89_; a similar process was used for N‐Myc_74–89_.

##### 2.1.7.7 General Points

Sequence data were obtained from UniProt knowledgebase [20] in FASTA format. Where necessary, databases and individual sequences were edited in R (version 4.2.0; R Core Team, 2022) using package seqinR (version 4.2‐16) [21].

##### 2.1.7.8 Human Recombinant Aurora Kinase A

The sequence of full‐length human Aurora‐A was obtained from UniProt accession O14965 on 16 August 2022. This sequence was modified by first making substitutions C290A and C393A, then removing residues 1–118, and finally substituting the three amino acid residues at the new N‐terminus (Lys‐Asn‐Glu) with Gly‐Ala‐Met.

##### 2.1.7.9 N‐Myc_73–89_

The sequence of full‐length N‐Myc was obtained from UniProt accession P04198 on 19 April 2023. Residues 73–89 were excised from the full‐length sequence.

##### 2.1.7.10 Potential Contaminants

The sequence of mature bovine serum albumin (amino acid residues 25–607 of the precursor) was obtained from UniProt accession P02769 on 13 June 2022. The Swiss‐Prot *E. coli* K12 proteome (4400 sequences, deemed representative of the proteome of the expression host) was obtained from UniProt on 23 August 2023. MaxQuant's database of potential contaminants (‘contaminants.fasta’) was edited to remove all bovine proteins. Three other proteins were also considered as potential contaminants (human recombinant BCL‐x_L_ [22], recombinant hDM2 [23] and human recombinant MCL‐1 [22]).

##### 2.1.7.11 Variable‐Modification Search

A variable‐modification search was performed in MaxQuant (version 2.1.4.0, Max Planck Institute of Biochemistry), using the built‐in Andromeda search engine [24]. A new modification, ‘NMyc7389AS (KSTY)’, was configured as follows: composition, C_100_H_139_O_31_N_21_S_2_ (2193.9387 Da); position, ‘anywhere’; type, ‘standard’; new terminus, ‘none’; specificities, K, S, T and Y. The sequence database contained human recombinant Aurora‐A, human recombinant BCL‐x_L_, recombinant hDM2, human recombinant MCL‐1, mature bovine serum albumin and the *E. coli* K12 proteome. Further potential contaminants were included by enabling ‘include contaminants’. For in silico digestion, the mode was ‘specific’, the enzyme was ‘trypsin/P’ and the maximum number of missed cleavages was two. The modifications were methionine oxidation (variable), ‘NMyc7389AS (KSTY)’ (variable) and cysteine carbamidomethylation (fixed). The maximum number of modifications per peptide was five. The maximum allowed charge was five.

##### 2.1.7.12 Cross‐Link Search

A cross‐link search was also performed in MaxQuant (version 2.1.0.0) using the Andromeda search engine in conjunction with the ‘MaxLynx’ module [25]. For the purpose of the search, probe adducts were conceptualized as N‐Myc_73–89_ amide joined to human recombinant Aurora‐A via a linker with the molecular formula C_7_H_4_O_3_S (replaces one hydrogen atom on N‐Myc_73–89_ amide and one hydrogen atom on human recombinant Aurora‐A). According to this model, a new cross‐link was configured as follows: linked composition, C_7_H_2_O_3_S; hydrolysed composition, C_7_H_4_O_4_S; specificity 1, ‘E’; position in peptide 1, ‘anywhere’; protein N‐term 1, enabled; protein C‐term 1, disabled; specificity 2, ‘KSTY’; position in peptide 2, ‘anywhere’; protein N‐term 2, enabled; protein C‐term 2, enabled; MS‐cleavable, disabled. The sequence database contained human recombinant Aurora‐A, N‐Myc_73–89_ and four potential contaminants (human recombinant Bcl‐x_L_, recombinant hDM2, human recombinant Mcl‐1 and mature bovine serum albumin). The ‘include contaminants’ option was disabled. For in silico digestion, the mode was ‘specific’, the enzyme was ‘trypsin/P’ and the maximum number of missed cleavages was three. The maximum peptide mass was 6500 Da. The modifications were methionine oxidation (variable), protein C‐terminal amidation (variable) and cysteine carbamidomethylation (fixed). The maximum number of modifications per peptide was five. The maximum allowed charge was five. The MaxLynx search was enabled by selecting the newly configured cross‐link, with default parameters. Peak refinement was enabled.

##### 2.1.7.13 Filtering and Visualization of Search Results

Hits from the MaxLynx search (crosslinkMsms.txt) were filtered using the following criteria: (i) hit must not be a decoy; (ii) posterior error probability ≤ 0.01; (iii) cross‐link product type must be ‘Inter‐protein link’; (iv) cross‐link must be between N‐Myc_73–89_ and human recombinant Aurora‐A; (v) cross‐linked amino acid residue of N‐Myc_73–89_ must be N‐terminal Glu; (vi) other modifications to N‐Myc_73–89_ must include C‐terminal amidation. The human recombinant Aurora‐A peptides that remained after filtering were mapped to the Aurora‐A reference sequence using an R script based on Preston and co‐authors' Script IV [26].

## 3 Results and Discussion

**FIGURE 1:**
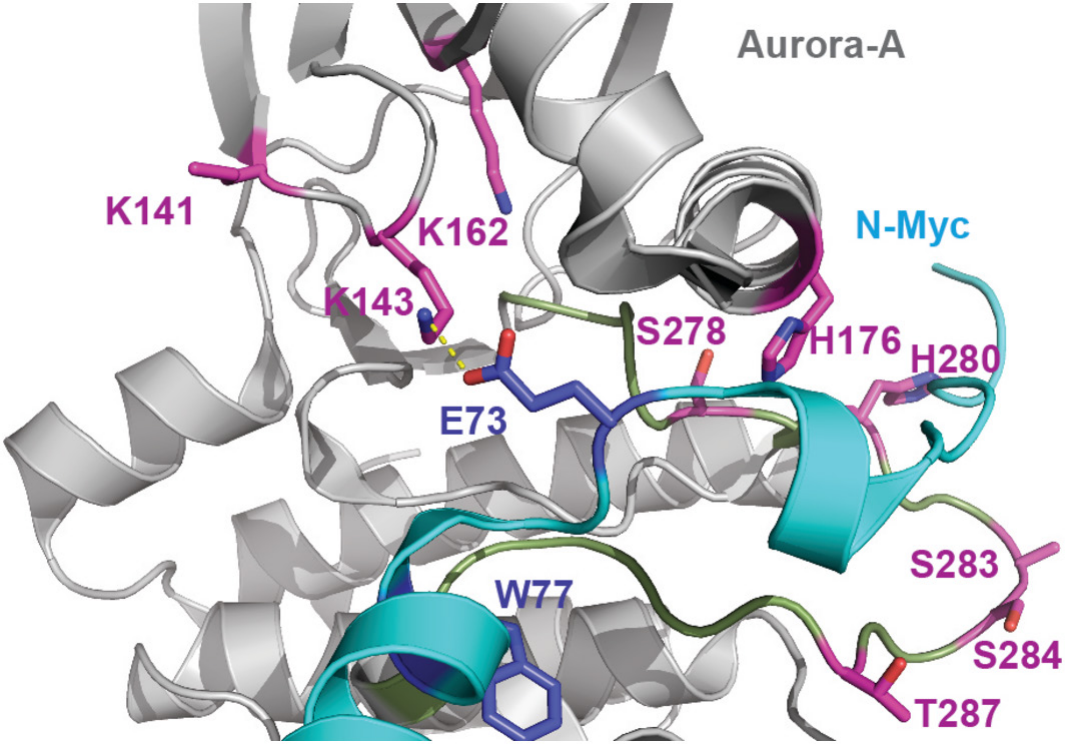
Magnified view of the N‐Myc (cyan)/Aurora‐A (grey) interaction (PDB: 5G1X) [13] at the Glu73^N‐Myc^‐Lys143^AurA^ salt bridge (charge‐reinforced contact shown as dashed yellow line). Side chains on Aurora‐A proximal to the salt bridge that can potentially be targeted with electrophilic sulfonyl fluoride warheads are highlighted as magenta sticks (note that Lys141^AurA^ and Ser283^AurA^ side chains are not fully resolved in the crystal structure). Hotspot side chains (dark blue) and the Aurora‐A A‐loop (limon) are also highlighted.

In the N‐Myc/Aurora‐A co‐crystal structure, there are no accessible cysteines at the PPI interface; however, the Glu73^N‐Myc^‐Lys143^AurA^ salt bridge appears to contribute to complex formation (Figure 1) [13]. Targeting lysine residues is desirable in the absence of a cysteine proximal to a ligand binding site [27, 28]. Moreover, ligands targeting surface‐exposed lysine residues on Aurora‐A have been described [29, 30]. Lysine reactivity is largely governed by the chemical environment and p*K*_a_ of the amino nitrogen, which behaves as a hard nucleophile. Under physiological conditions, most surface‐exposed lysine side chains are protonated (p*K*_a_ **~** 10.4) and therefore are weakly nucleophilic; although buried lysines can have p*K*_a_ values as low as 5.7 [8]. Hard sulfonyl fluoride electrophiles have emerged as useful reactive probes in chemical biology and molecular pharmacology since they possess an ideal balance of biocompatibility, aqueous stability and protein reactivity [31]. Sulfonyl fluorides are known to modify a range of side chains including lysine, as well as tyrosine, serine, threonine and, to a lesser extent, cysteine and histidine [31, 32, 33, 34]. Importantly, however, their reactivity appears to be context‐specific, depending on both the environment of the nucleophile (i.e., the nucleophilicity) and the effective molarity of the reaction inferred through reversible, noncovalent affinity of the probe [35]. Finally, aryl sulfonyl fluorides have proven successful as warheads in peptidomimetic inhibitors of PPIs [32, 33, 36, 37]. Lys143^AurA^ seemed a suitable residue to target in this preliminary study given its proximity to the N‐Myc helical binding epitope; however, a number of nucleophilic lysine, histidine and serine/threonine residues are nearby, including within the flexible activation loop of Aurora‐A, which might also react with electrophiles introduced to the N‐Myc sequence. Recent reports on the reaction of aryl sulfonyl fluorides are particularly pertinent in this respect [32, 33, 34]. We therefore sought in this work to covalently target Lys143^AurA^, despite it being surface‐exposed and cognisant of the potential to react with other proximal residues, using N‐Myc‐based peptides armed with an electrophilic sulfonyl fluoride group.

**SCHEME 1:**
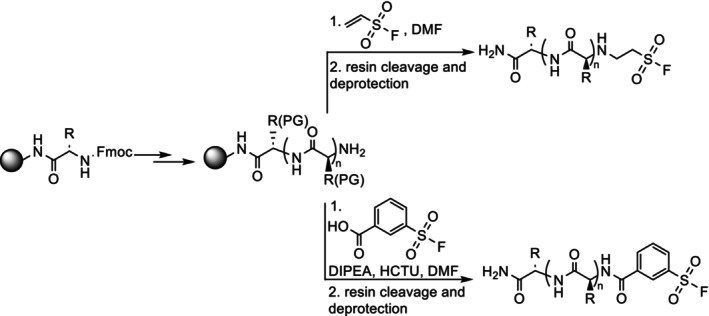
Outline of the synthetic approach to the preparation of sulfonyl fluoride appended peptides by solid‐phase peptide synthesis.

To covalently target Lys143^AurA^ the replacement of Glu73^N‐Myc^ with an electrophilic warhead seemed promising, as this would place the reactive warhead in proximity to Lys143^AurA^. However, the most effective covalent probes tend to combine low reactivity with high reversible binding affinity [5]; thus, retaining the Glu73^N‐Myc^ residue was considered potentially advantageous in terms of noncovalent affinity. Given the noncovalent interaction between Glu73^N‐Myc^ and Lys143^AurA^, we considered this may bring the reactive electrophile into proximity with the Lys143^AurA^ side chain to encourage covalent modification. A series of N‐Myc_73–89_ and N‐Myc_74–89_ peptides were prepared using solid‐phase peptide synthesis (Scheme 1, see ESI for experimental details and characterization), bearing ESF and ASF electrophiles at their *N*‐termini (Table 2). Using this approach, the sulfonyl fluorides could readily be introduced by conjugation to the peptide N‐terminus prior to global deprotection and resin cleavage.

**TABLE 2:** Sequences and inhibitory parameters of N‐Myc peptides bearing *N*‐terminal sulfonyl fluoride warheads.

Peptide	Sequence	IC_50_ (μM)	*K*_i_ (μM)	*K*_inact_ (×10^−5^ s^−1^)	*K*_inact_/*K*_i_ (M^−1^ s^−1^)
N‐Myc_73–89_	EPPSWVTEMLLENELWG	179 ± 19	n.d.	n.d.	n.d.
N‐Myc_73–89 ESF_	EPPSWVTEMLLENELWG	n.d.	n.d.	n.d.	n.d.
N‐Myc_74–89 ESF_	PPSWVTEMLLENELWG	n.d.	n.d.	n.d.	n.d.
N‐Myc_73–89 ASF_	EPPSWVTEMLLENELWG	33 ± 2	62 ± 7	9.8 ± 0.5	1.6 ± 0.2
N‐Myc_74–89 ASF_	PPSWVTEMLLENELWG	118 ± 9	313 ± 85	110 ± 25	3.5 ± 1.2
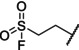		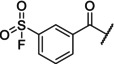			

*Note:* IC_50_ values given as the mean value and corresponding standard deviation (SD) determined from competition FA assays (*n* = 3, 50‐nM FAM‐Ahx‐N‐Myc_61–89_, 15‐μM Aurora‐A_122–403 C290A/C393A_, 25‐mM Tris, 150‐mM NaCl, 5‐mM MgCl_2_ and pH 7.5), k_inact_/K_I_ second order rate constants were calculated based on k_inact_ and K_I_ values determined via kinetic mass‐spectrometry analyses.

Despite the successful synthetic introduction of ESF to the *N*‐termini of N‐Myc_73–89_ and N‐Myc_74–89_ peptides (see ESI), the electrophilic moiety of both ESF‐bearing peptides hydrolysed during preparative HPLC. However, both peptides bearing ASF fragments showed better aqueous stability during purification, and both N‐Myc_73–89 ASF_ and N‐Myc_74–89 ASF_ were isolated with no issue.

**FIGURE 2:**
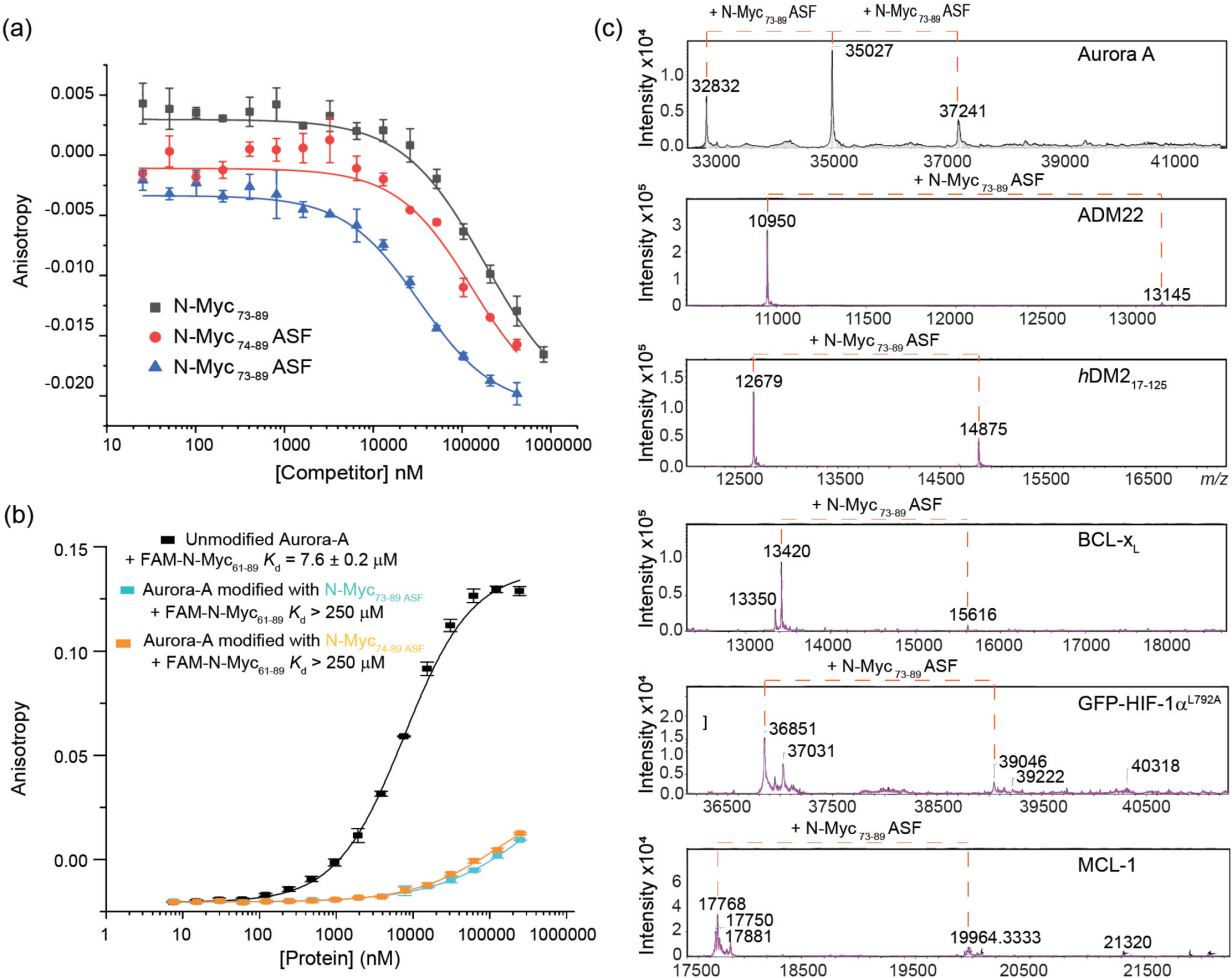
Investigating covalent peptidomimetics for Aurora‐A binding and modification. (a) Competition FA curves ([tracer] = 50 nM, 25 mM Tris, 150 mM NaCl, 5 mM MgCl_2_, pH 7.5, 2 h; all data points represent the mean of three technical replicates; error bars indicate SD). (b) Direct FA curves for Aurora‐A and N‐Myc_73–89 ASF_ or N‐Myc_74–89 ASF_ modified Aurora‐A ([tracer] = 50 nM, 25 mM Tris, 150 mM NaCl, 5 mM MgCl_2_, pH 7.5, 2 h; all data points represent the mean of three technical replicates; error bars indicate SD). (c) Mass spectrometry analyses of N‐Myc_73–89 ASF_ in the presence of Aurora‐A, ADM22, *h*DM2_17–125_, BCL‐x_L_, GFP‐HIF‐1α_L792A_ and MCL‐1 (measured after 3 h, [peptide] = 45 μM [protein] = 90 μM, 25 mM Tris, 150 mM NaCl, 5 mM MgCl_2_ and pH 7.5).

We first performed competition FA assays with FAM‐Ahx‐N‐Myc_61–89_ (referred to henceforth as tracer) and Aurora‐A_122–403 C290A/C393A_ (referred to henceforth as Aurora‐A). These FA competition assays revealed that the two ASF‐bearing peptides have a difference in inhibitory potency for the displacement of the tracer from Aurora‐A, with IC_50_ values for N‐Myc_73–89 ASF_ = 33 ± 2 μM and N‐Myc_74–89 ASF_ = 118 ± 9 μM (Figure 2a, Table 2). These data suggest in the case of N‐Myc_73–89 ASF_ that the ASF warhead has a beneficial effect (IC_50_ N‐Myc_73–89_ = 179 ± 19 μM) and supports the hypothesis that retention of Glu73^N‐Myc^ enhances reversible binding of N‐Myc_73–89 ASF_ to Aurora‐A, facilitating its covalent modification. To further demonstrate that the ASF‐derived peptides act as covalent inhibitors, we prepared Aurora‐A modified with N‐Myc_73–89 ASF_ or N‐Myc_74–89 ASF_ and carried out direct titration with the tracer (Figure 2b). In the absence of any modification, the tracer bound to Aurora‐A with a *K*_d_ of 7.6 μM, whereas following modification, tracer binding was much weaker (*K*_d_ > 250 μM), indicating its binding had been occluded as a consequence of protein modification.

To investigate the ability of N‐Myc_73–89 ASF_ to covalently modify Aurora‐A, mass spectrometry analyses of the kinase in the presence and absence of the peptide were then carried out. N‐Myc_73–89 ASF_ was added to Aurora‐A at a 2:1 (peptide:protein) molar stoichiometry and, following a 3‐h incubation period, significant formation of covalently modified Aurora‐A was observed (Figure 2c), with an increase in molecular weight (*M*_W_) from 32,832 to 35,027 Da, consistent with the formation of sulfonyl‐peptide adduct. A second adduct (*M*_W_ = 37,241) was also observed, consistent with the double modification of Aurora‐A. To probe the selectivity of N‐Myc_73–89 ASF_ for Aurora‐A, MS analysis was carried out with five proteins: an Adhiron (ADM22) [38], *h*DM2_17–125_ [39], BCL‐x_L_ [38], GFP‐HIF‐1α_L792A_ [40] and MCL‐1 [38] (Figures 2c and S1–S6 for time‐dependent data). Although, in some cases, small amounts of modified protein could be observed (most significantly for hDM2), the extent of covalent modification of these proteins was significantly less than that of Aurora‐A, indicating that N‐Myc_73–89 ASF_ exhibits a degree of selectivity for covalent modification of Aurora‐A and that this modification is therefore recognition‐directed.

To further investigate the ability of the covalent peptides to modify Aurora‐A, each probe was incubated (for 2 h) in the presence of Aurora‐A at a 2:1‐M stoichiometry and analyzed by MS (Figure S7). It was hypothesized that the high protein and peptide concentrations used during the initial analysis (45 and 90 μM, respectively) may have encouraged non‐specific reactions. Lower protein and peptide concentrations of 10 and 20 μM were used, respectively. Unsurprisingly, given the reduced incubation period and lower protein/peptide concentrations, N‐Myc_73–89 ASF_ modified Aurora‐A to a lesser extent than observed previously. Nonetheless, considerable formation of the expected product mass ion was detected, along with minor formation of the double mass adduct. Despite N‐Myc_74–89 ASF_ displaying reduced potency in the initial competition FA analysis compared to N‐Myc_73–89 ASF_, this less potent variant modified Aurora‐A to a greater extent by MS analysis (further discussed below).

**FIGURE 3:**
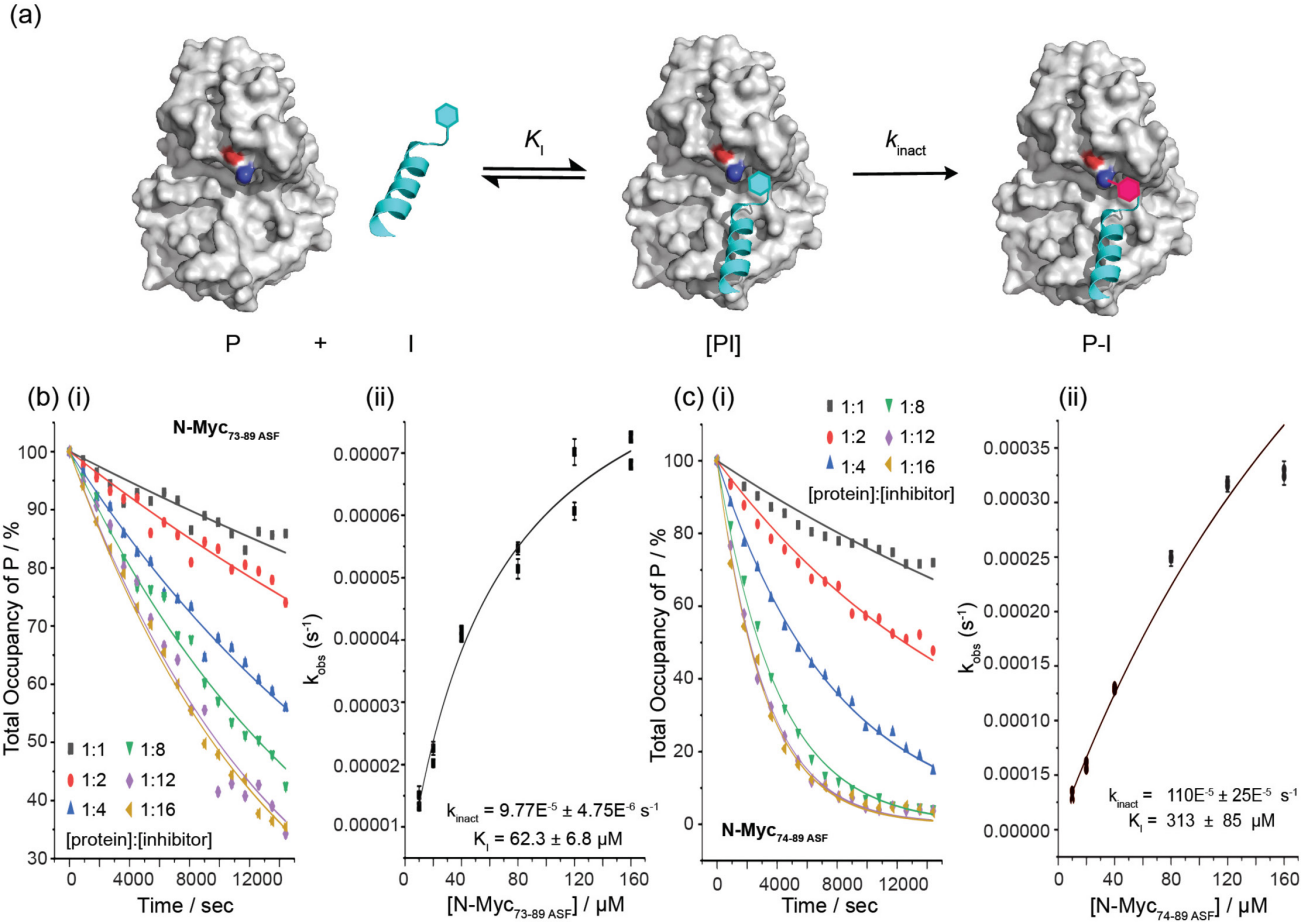
Determining *k*_obs_, *k*_inact_ and *K*_I_ values. (a) A two‐step mechanism of covalent protein modification: P is the target protein with an exposed nucleophilic side chain (blue region), I is the inhibitor bearing an electrophilic warhead (cyan hexagon), [PI] is the reversibly formed protein inhibitor complex and P‐I is the covalently modified protein. The formation of a covalent bond between the target protein and inhibitor is shown through the colour change of the warhead from cyan to deep pink. The first step (reversible binding) is defined by the binding constant *K*_I_, and the maximum rate of the second step (irreversible covalent reaction) is defined by *k*_inact_. (b) Plots of % occupancy of unmodified Aurora‐A against time upon reaction with N‐Myc_73–89 ASF_ as a function of protein:inhibitor ratio; these data (fit based on 2 and 4 h) were used to obtain k_obs_ values, which were plotted against [N‐Myc_73–89 ASF_] to determine *k*_inact_ and *K*_I_ values (Aurora‐A 10 μM; 25 mM Tris, 150 mM NaCl, 5 mM MgCl_2_ and pH 7.5). (c) Plots of % occupancy of unmodified Aurora‐A against time upon reaction with N‐Myc_74–89 ASF_ as a function of protein:inhibitor ratio, with the data points used to obtain *k*_obs_ values, which were plotted against [N‐Myc_74–89_ ASF] to determine *k*_inact_ and *K*_I_ values (Aurora‐A 10 μM; 25 mM Tris, 150 mM NaCl, 5 mM MgCl_2_ and pH 7.5).

The modification of a target protein by a covalent inhibitor is a two‐step process (Figure 3a). Firstly, the inhibitor (I) binds to the protein (P) to form a reversible complex ([PI]), and then covalent bond formation occurs to give the product (P‐I). An accurate measure of the effectiveness of covalent inhibitors must account for both steps involved in the modification of the target protein [41]. *k*_inact_ and *K*_I_ values were determined using MS analysis (Figures 3b and S8 and S9); by comparing the relative areas of the observed peaks, it was possible to estimate total occupancy of the different protein states (P, P‐I, I‐P‐I, etc.) over time at a range of different inhibitor concentrations (i.e., protein:inhibitor stoichiometries). The total occupancies of different protein states over a 4‐h period obtained for different concentrations of N‐Myc_73–89 ASF_ and N‐Myc_74–89 ASF_ in the presence of Aurora‐A (Figures S8 and S9) were used to determine the % occupancy of P‐I with time. In the case of N‐Myc_73–89 ASF_, small amounts of doubly modified Aurora‐A (i.e., I‐P‐I) were also observed, especially at protein:inhibitor stoichiometries of 1:8 and above (see ESI Figure S8). The formation of I‐P‐I adducts was more pronounced in the presence of N‐Myc_74–89 ASF_. Fitting the data points for change in the occupancy of P over time (i.e., accounting for all modifications) provided more accurate values for *k*_obs_ (than for individual modifications) at different inhibitor concentrations (Figure 3b). The resulting k_obs_ values were plotted against inhibitor concentration, and the data points were used to determine *k*_inact_ and *K*_I_ values. To further understand the error in determining k_obs_ (and hence, *k*_inact_/*K*_I_ values), the change in occupancy of P over 2 h was also used to obtain *k*_obs_. The combined *k*_obs_ values were plotted against inhibitor concentration (Figure 3b) and used to determine *k*_inact_ = 9.8 × 10^−5^ ± 0.5 × 10^−5^ s^−1^ and K_I_ = 62 ± 7 μM values for N‐Myc_73–89 ASF_, with a *k*_inact_/*K*_I_ value of 1.6 ± 0.2 M^−1^ s^−1^. For N‐Myc_74–89 ASF_, the occupancy of the unmodified Aurora‐A construct decreased more rapidly. The determined *k*_obs_ values were plotted against inhibitor concentration and used to determine the *k*_inact_ (110 × 10^−5^ ± 25 × 10^−5^ s^−1^), *K*_I_ (313 ± 85 μM) values and hence *k*_inact_/*K*_I_ value (3.5 ± 1.2 M^−1^ s^−1^) (Table 2). Both peptides exhibit low *k*_inact_/*K*_I_ values in comparison to covalent drugs (e.g., bearing acrylamide for EGFR) [42] and covalent peptidomimetics (e.g., bearing arylsulfonyl fluoride for MCL‐1) [37]; in this instance, reflecting both low affinity and low reactivity, although high affinity peptidomimetics (e.g., for the bacterial sliding clamp) bearing weak electrophilic groups (e.g., acrylamide or chloroacetamide) have been shown to elicit similarly low *k*_inact_/*K*_I_ values [43]. The linearity of k_obs_ against inhibitor concentration plots provides an approximate measure of the dependence of the rate of modification on noncovalent binding affinity. A non‐specific covalent inhibitor (or an inhibitor with a very large *K*_I_ value) would yield a linear plot with slope equal to *k*_inact_/*K*_I_ [27, 41]. Thus, N‐Myc_73–89 ASF_ shows clear evidence of recognition‐directed labelling. In contrast, N‐Myc_74–89 ASF_ modified Aurora‐A rapidly; however, the plot of k_obs_ against inhibitor concentration indicates that N‐Myc_74–89 ASF_ may be a non‐specific inhibitor with low noncovalent affinity, which is consistent with the observed *K*_I_ value. It is not obvious why the lower affinity peptide (N‐Myc_74–89 ASF_) would confer more rapid and extensive modification of Aurora‐A. Peptides with higher reversible affinity would be expected to modify the protein to a greater extent due to increased effective molarity. There are several possible explanations. Firstly, N‐Myc_74–89 ASF_ could covalently react with a different, possibly more nucleophilic Aurora‐A side chain than N‐Myc_73–89 ASF_. There are a number of side chains proximal to Lys143^AurA^ with which an electrophilic sulfonyl fluoride could react (see Figure 1). It is conceivable that N‐Myc_74–89 ASF_ reacts with the intended Lys143^AurA^, whilst N‐Myc_73–89 ASF_ targets His176^AurA^, or another side chain located in the flexible kinase A‐loop. In addition, Glu73^AurA^ may suppress the reactivity of the ASF warhead in N‐Myc_73–89 ASF_. Alternatively, a highly efficient specific inhibitor can produce a linear plot akin to that of a non‐specific inhibitor [41]; thus, N‐Myc_74–89 ASF_ could just be more efficient than N‐Myc_73–89 ASF_. Based on the N‐Myc/Aurora‐A structure, the ASF fragment would be expected to be closer to Lys143^AurA^ when connected to Pro74^N‐Myc^ as opposed to Glu73^N‐Myc^.

We next attempted to identify the site of protein labelling using proteolysis followed by MS/MS experiments. Unfortunately, these experiments proved challenging—whilst we were able to obtain good sequence coverage for unmodified Aurora‐A, for covalently modified Aurora‐A, the standard peptide search algorithms were unable to provide conclusive identification of the modified peptides in the MS–MS spectra, despite clear differences in the MS1 chromatograms. This could arise due to the size of the modified Aurora‐A peptide fragments (bearing the N‐Myc modification which in itself could be difficult to identify due to fragmentation), or their hydrophobicity. Manual inspection of the spectra allowed identification of a possible modification to a tyrosine in the N‐lobe of Aurora‐A; however, these data should be treated with caution given the low intensity in the MS–MS spectra (see Supporting Information for further discussion).

## 4 Conclusions

In this work, we developed a covalent peptidomimetic for inhibition of the N‐Myc/Aurora‐A interaction. We did so by using an ASF electrophile conjugated to N‐Myc peptides. The resultant peptidomimetics were shown to act as more potent PPI inhibitors than their parent noncovalent sequences and labelled Aurora‐A selectively and in a recognition‐directed manner as demonstrated by kinetic labelling analyses. Whilst we were unable to validate the design in terms of the intended labelling site, all other analyses were consistent with orthosteric inhibition of the N‐Myc/Aurora‐A interaction and labelling on a nucleophilic residue proximal to this binding site. Despite this, both peptides exhibit low *k*_inact_/*K*_I_ values, which arise due to a combination of low potency and covalent reactivity. A systematic library of peptides where the position in the sequence and linker chemistry of the sulfonyl fluoride are varied, or alternative electrophilic groups employed that react with different amide acid side chains on the surface of Aurora‐A, will be needed to generate a more potent ligand. Future studies will be directed towards these goals. More broadly, whilst active site covalent inhibitors have seen a rapid rise in prominence, covalent PPI inhibitors and covalent peptidomimetics are less well developed; thus, these experiments further contribute to growing efforts in this area.

## Author Contributions

A.J.W. conceived and designed the research programme, with the input of M.H.W., R.B. and S.L.W. R.S.D. and D.G. designed the studies and performed the research. G.W.P. carried out site‐mapping of covalent modifications. A.J.W. and R.S.D. wrote the manuscript, which was edited into its final form through contributions from all authors.

## Conflicts of Interest

The authors declare no conflicts of interest.

## Supporting information


**Figure S1:** Mass spectrometry analyses of N‐Myc_73–89 ASF_ in the presence of Aurora‐A at 0, 1 and 3 h ([peptide] = 45 μM] [protein] = 90 μM, 25‐mM Tris, 150‐mM NaCl, 5‐mM MgCl_2_ and pH 7.5).
**Figure S2:** Mass spectrometry analyses of N‐Myc_73–89 ASF_ in the presence of ADM22 at 0, 1 and 3 h ([peptide] = 45 μM] [protein] = 90 μM, 25‐mM Tris, 150‐mM NaCl, 5‐mM MgCl_2_ and pH 7.5).
**Figure S3:** Mass spectrometry analyses of N‐Myc_73–89 ASF_ in the presence of *h*DM2 at 0, 1 and 3 h ([peptide] = 45 μM] [protein] = 90 μM, 25‐mM Tris, 150‐mM NaCl, 5‐mM MgCl_2_ and pH 7.5).
**Figure S4:** Mass spectrometry analyses of N‐Myc_73–89 ASF_ in the presence of BCL‐x_L_ at 0, 1 and 3 h ([peptide] = 45 μM] [protein] = 90 μM, 25‐mM Tris, 150‐mM NaCl, 5‐mM MgCl_2_ and pH 7.5).
**Figure S5:** Mass spectrometry analyses of N‐Myc_73–89 ASF_ in the presence of GFP‐HIF‐1α^L792A^ at 0, 1 and 3 h ([peptide] = 45 μM] [protein] = 90 μM, 25‐mM Tris, 150‐mM NaCl, 5‐mM MgCl_2_ and pH 7.5).
**Figure S6:** Mass spectrometry analyses of N‐Myc_73–89 ASF_ in the presence of MCL‐1 at 0, 1 and 3 h ([peptide] = 45 μM] [protein] = 90 μM, 25‐mM Tris, 150‐mM NaCl, 5‐mM MgCl_2_ and pH 7.5).
**Figure S7:** Mass spectrometry analyses of Aurora‐A (10 μM) incubated with (a) 20‐μM N‐Myc_73–89 ASF_ and (b) 20‐μM N‐Myc_74–89_ ASF after 2 h (25‐mM Tris, 150‐mM NaCl, 5‐mM MgCl_2_ and pH 7.5).
**Figure S8:** Plots of the % occupancy of the different protein states (P, P‐I, I‐P‐I, etc.) over time for Aurora‐A (10 μM) in the presence of N‐Myc_73–89 ASF_ at protein:inhibitor stoichiometries of (a) 1:1, (b) 1:2, (c) 1:4, (d) 1:8, (e) 1:12 and (f) 1:16 (25‐mM Tris, 150‐mM NaCl, 5‐mM MgCl_2_ and pH 7.5).
**Figure S9:** Plots of the % occupancy of the different protein states (P, P‐I, I‐P‐I, etc.) over time for Aurora‐A (10 μM) in the presence of N‐Myc_74–89 ASF_ at protein:inhibitor stoichiometries of (a) 1:1, (b) 1:2, (c) 1:4, (d) 1:8, (e) 1:12 and (f) 1:16 (25‐mM Tris, 150‐mM NaCl, 5‐mM MgCl_2_ and pH 7.5).
**Figure S10:** (a) Representative MS2 spectra for the fragmentation of the Myc_73–89 ASF_ labelled _no_FGNVYLAR_no_ fragment; (b) LC chromatograms corresponding to the ions from MS1 spectra corresponding to Aurora‐A peptides in the (i) Myc_73–89 ASF_ labelled and (ii) Myc_73–89 ASF_ unlabelled samples.
**Table S1:** Protein HRMS data.

## Data Availability

All experimental procedures and relevant data are included in the electronic Supporting Information ESI.
