# Supplementary material for: Covalent Peptide‐Based N‐Myc/Aurora‐A Inhibitors Bearing Sulfonyl Fluoride Warheads
Source: J Pept Sci. 2026 Feb 3;32(3):e70086. doi: 10.1002/psc.70086 (PMC12867955; doi:10.1002/psc.70086)

**Peptide-based N-Myc/Aurora-A Inhibitors** **bearing Sulfonyl Fluoride Warheads**

Robert S. Dawber^1^, Diana Gimenez,^2^ George W. Preston,^1,4^ Richard Bayliss,^3,4^ Megan H. Wright,^1,4^ Stuart L. Warriner,^1,4^ Andrew J. Wilson^1,2,4^

^1^School of Chemistry, University of Leeds, Woodhouse Lane, Leeds LS2 9JT, UK

^2^School of Chemistry, University of Birmingham, Edgbaston, B15 2TT, UK

^3^School of Molecular and Cellular Biology, University of Leeds, Woodhouse Lane, Leeds LS2 9JT, UK

^4^Astbury Centre for Structural Molecular Biology, University of Leeds, Woodhouse Lane, Leeds LS2 9JT, UK

**SUPPORTING INFORMATION**

Contents

[**Suplementary Data and Figures** 2](#_Toc212708942)

[**Attempted Site Mapping of Covalent Modification** 8](#_Toc212708943)

[**Modified Protein Masses** 9](#_Toc212708944)

[**Peptide Characterization Data** 11](#_Toc212708945)

# **Suplementary Data and Figures**


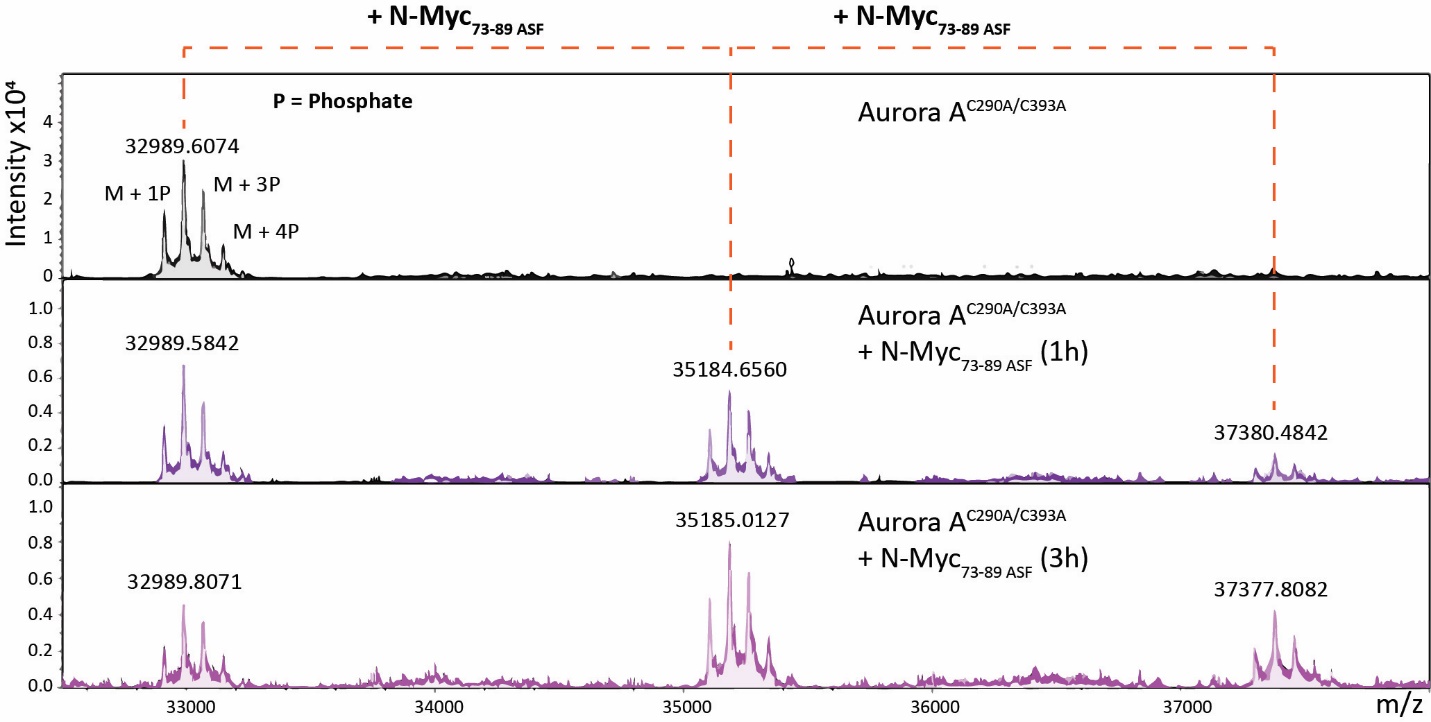


**Figure S1**. Mass Spectrometry analyses of N-Myc_73-89 ASF_ in the presence of Aurora A at 0, 1 and 3 hrs ([peptide] = 45 μM] [protein] = 90 μM, 25 mM Tris, 150 mM NaCl, 5 mM MgCl_2_ and pH 7.5).


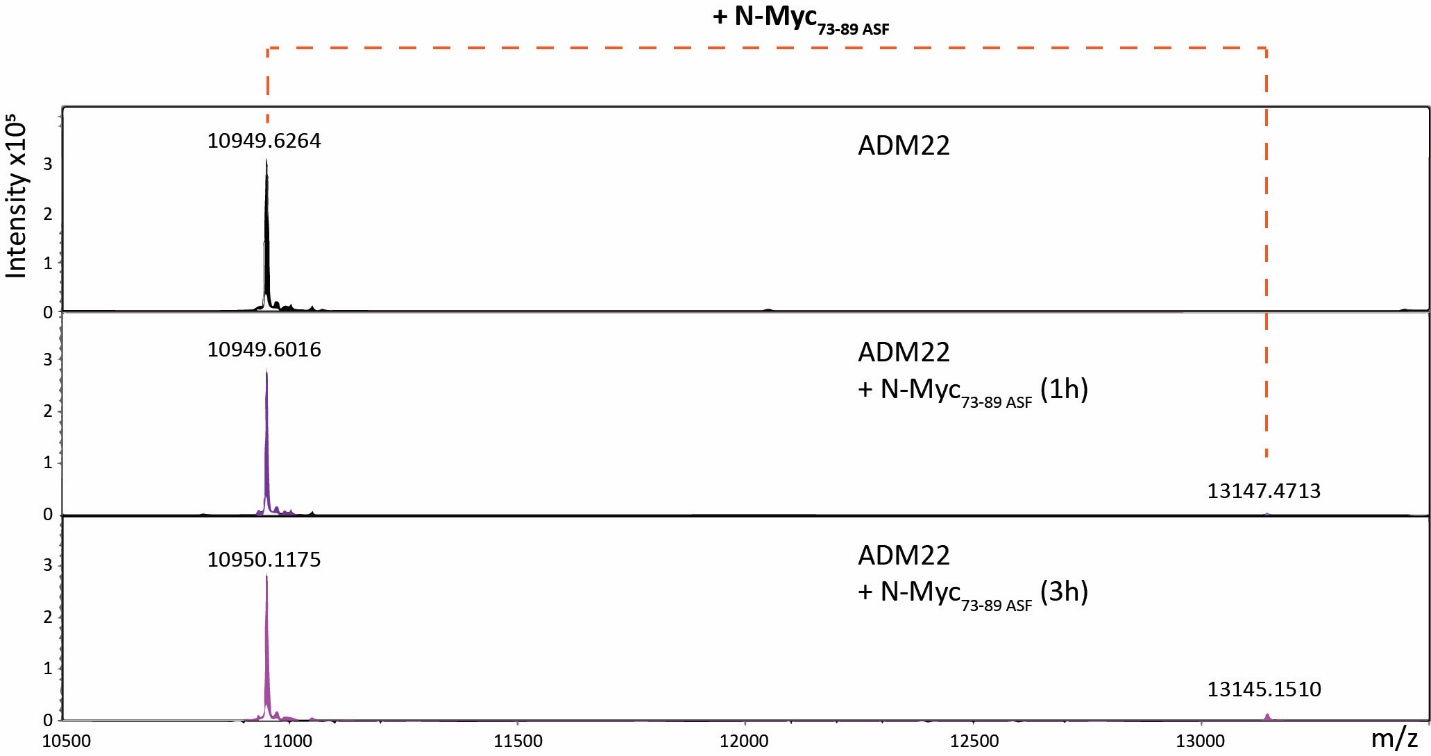


**Figure S2**. Mass Spectrometry analyses of N-Myc_73-89 ASF_ in the presence of ADM22 at 0, 1 and 3 hrs ([peptide] = 45 μM] [protein] = 90 μM, 25 mM Tris, 150 mM NaCl, 5 mM MgCl_2_ and pH 7.5).


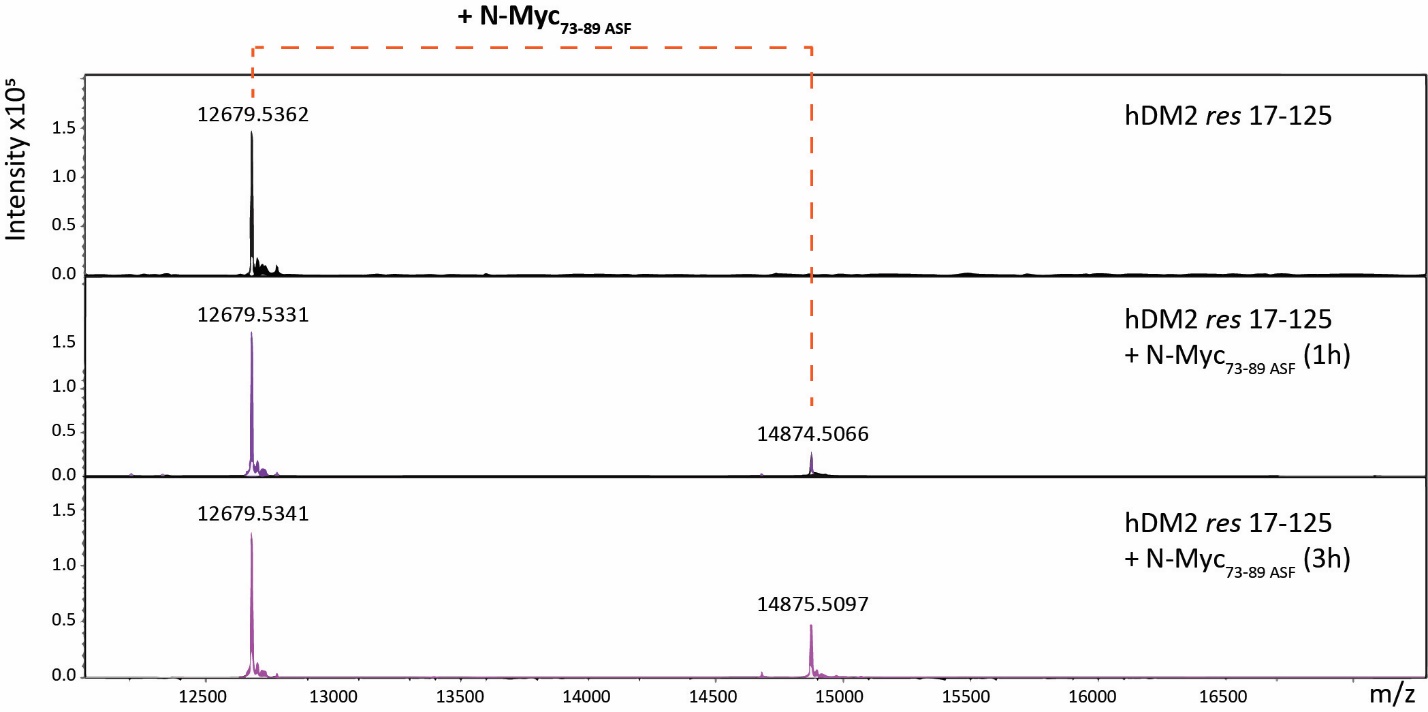


**Figure S3**. Mass Spectrometry analyses of N-Myc_73-89 ASF_ in the presence of hDM2 at 0, 1 and 3 hrs ([peptide] = 45 μM] [protein] = 90 μM, 25 mM Tris, 150 mM NaCl, 5 mM MgCl_2_ and pH 7.5).


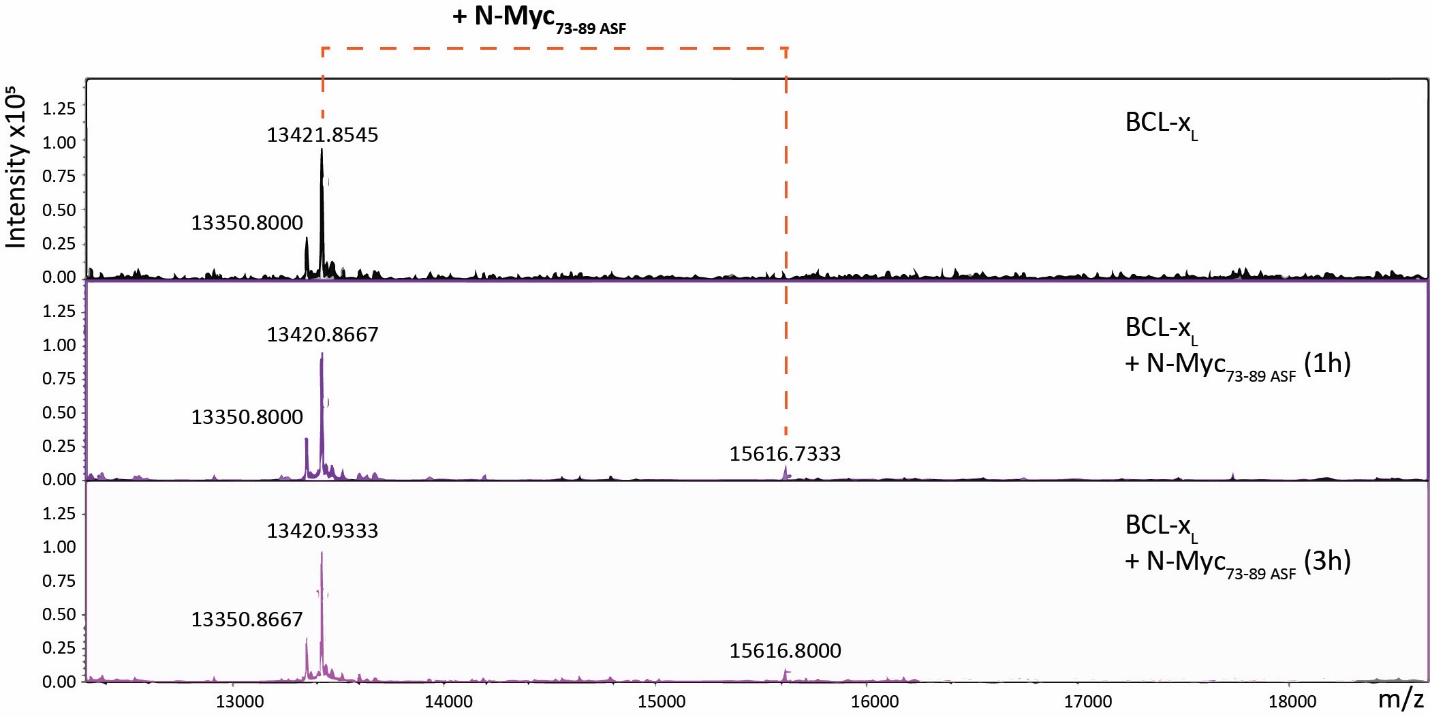


**Figure S4**. Mass Spectrometry analyses of N-Myc_73-89 ASF_ in the presence of BCL-x_L_ at 0, 1 and 3 hrs ([peptide] = 45 μM] [protein] = 90 μM, 25 mM Tris, 150 mM NaCl, 5 mM MgCl_2_ and pH 7.5).


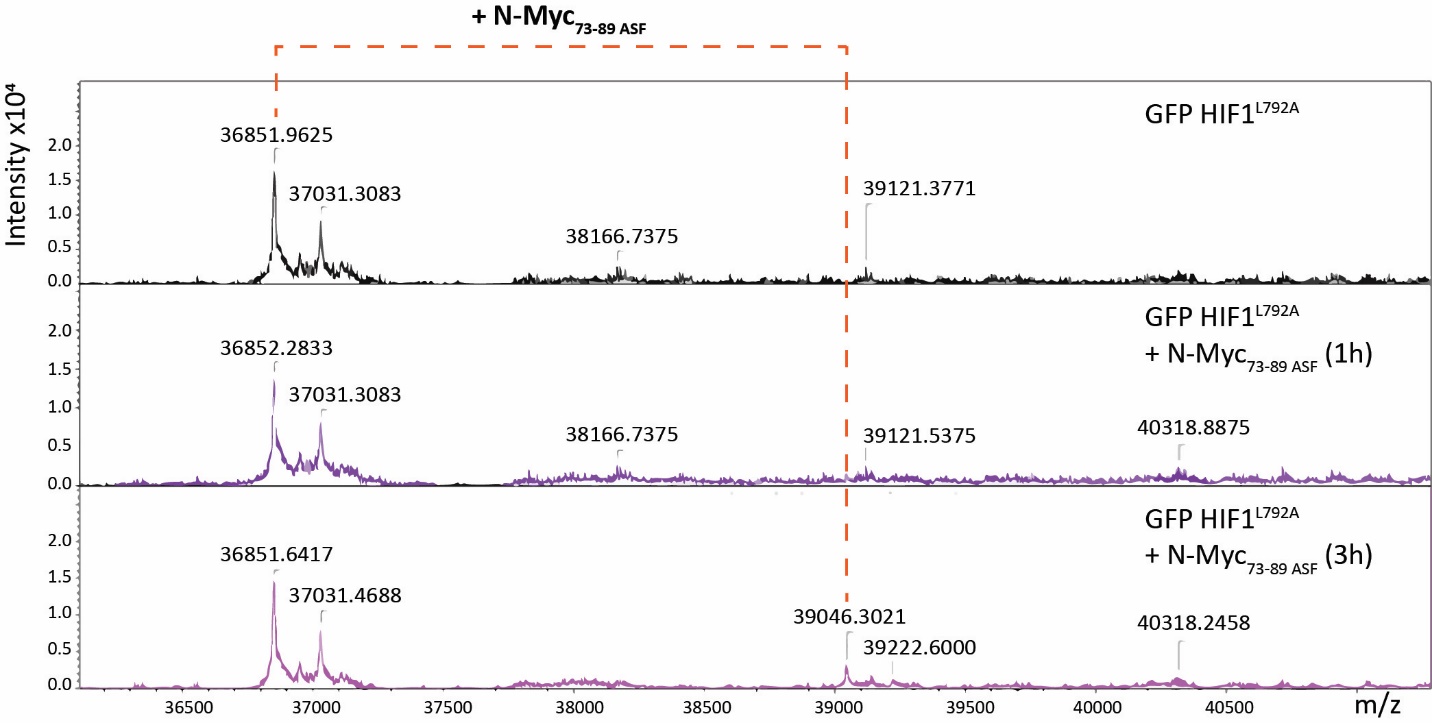


**Figure S5**. Mass Spectrometry analyses of N-Myc_73-89 ASF_ in the presence of GFP-HIF-1α^L792A^ at 0, 1 and 3 hrs ([peptide] = 45 μM] [protein] = 90 μM, 25 mM Tris, 150 mM NaCl, 5 mM MgCl_2_ and pH 7.5).


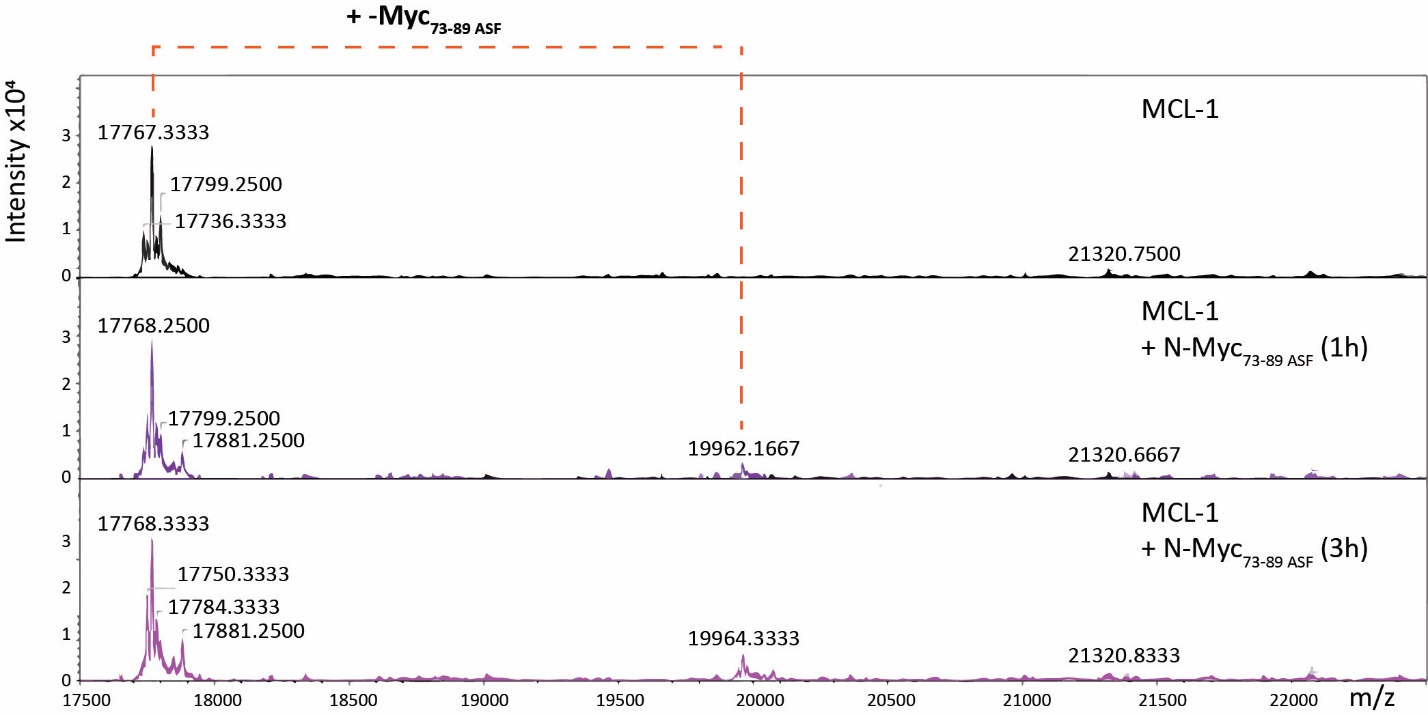


**Figure S6**. Mass Spectrometry analyses of N-Myc_73-89 ASF_ in the presence of MCL-1 at 0, 1 and 3 hrs ([peptide] = 45 μM] [protein] = 90 μM, 25 mM Tris, 150 mM NaCl, 5 mM MgCl_2_ and pH 7.5).


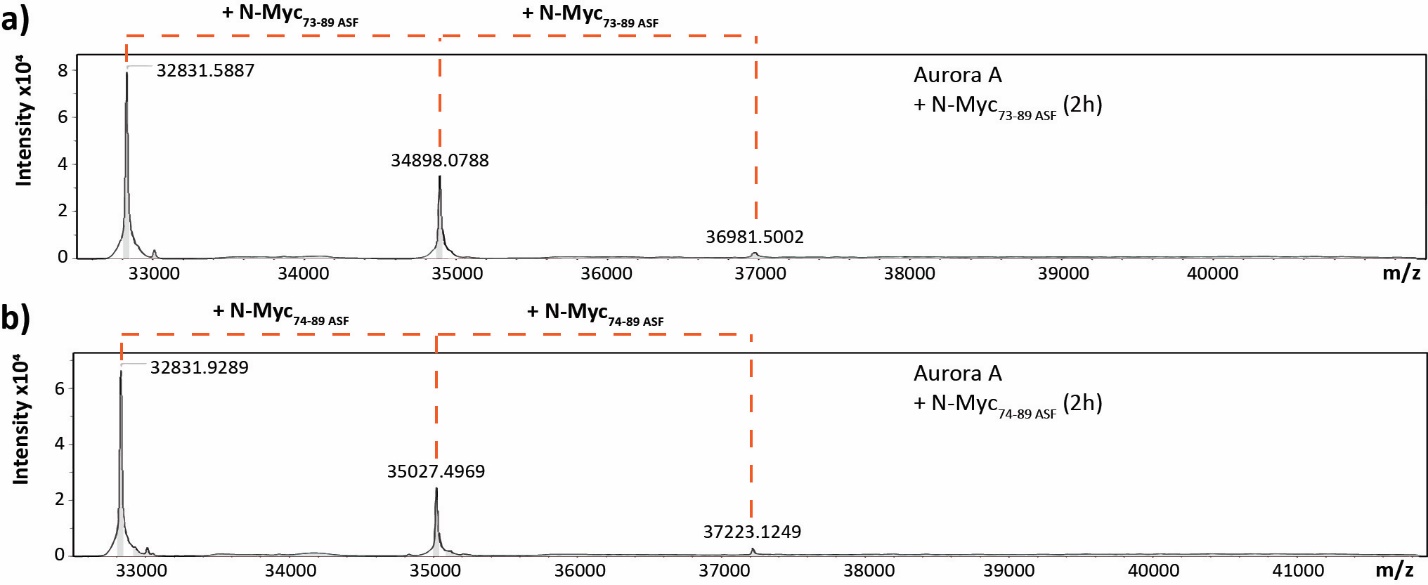


Figure S7: Mass Spectrometry analyses of Aurora A (10 μM) incubated with (a) 20 μM N-Myc_73-89 ASF_, (b) 20 μM N-Myc _74-89_ ASF after 2 hrs (25 mM Tris, 150 mM NaCl, 5 mM MgCl_2_, pH 7.5)


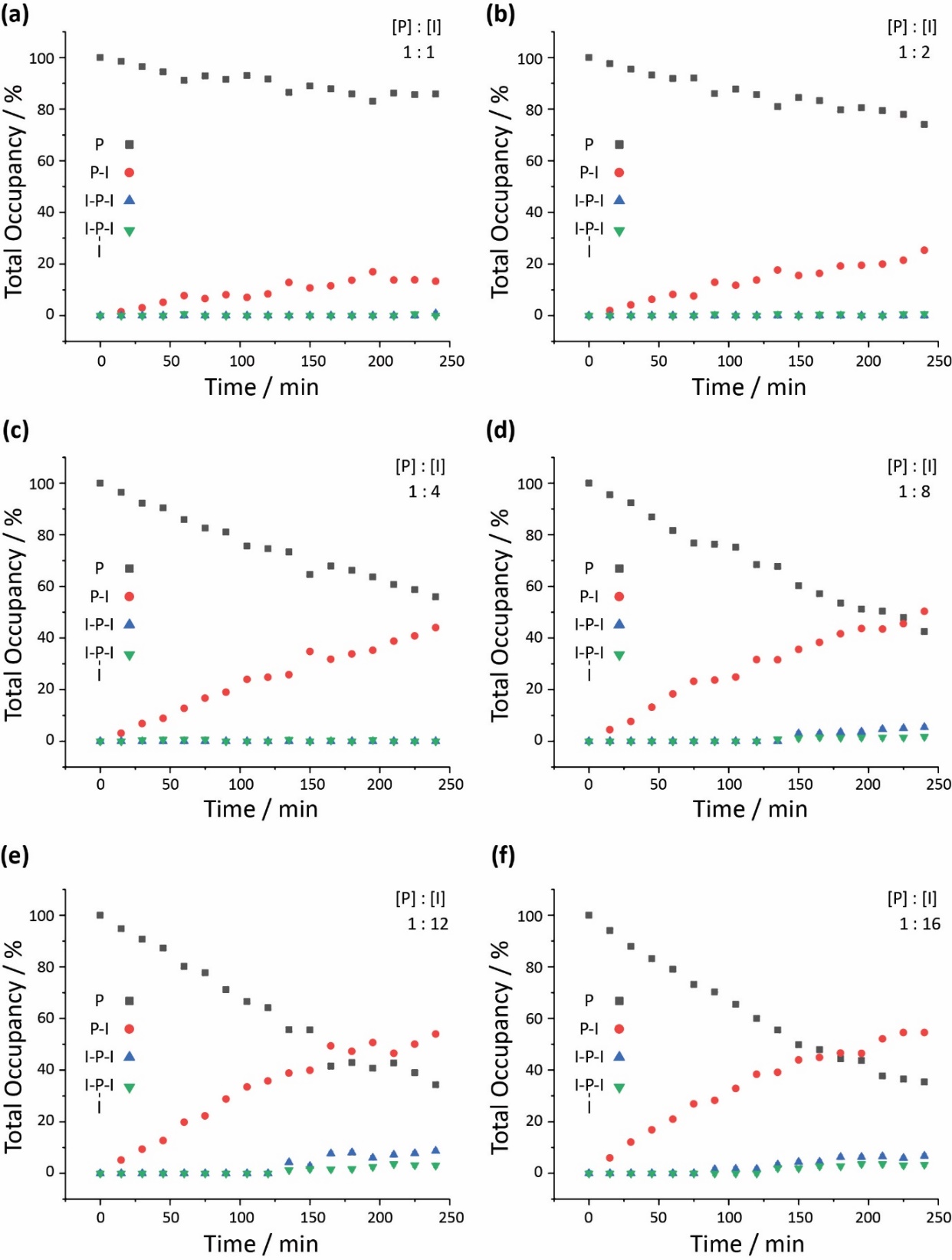


**Figure S8**. Plots of the % occupancy of the different protein states (P, P-I, I-P-I, etc.) over time for Aurora A (10 μM) in the presence of N-Myc_73-89 ASF_ at protein:inhibitor stoichiometries of (a) 1:1, (b) 1:2, (c) 1:4, (d) 1:8, (e) 1:12 and (f) 1:16 (25 mM Tris, 150 mM NaCl, 5 mM MgCl_2_, pH 7.5).


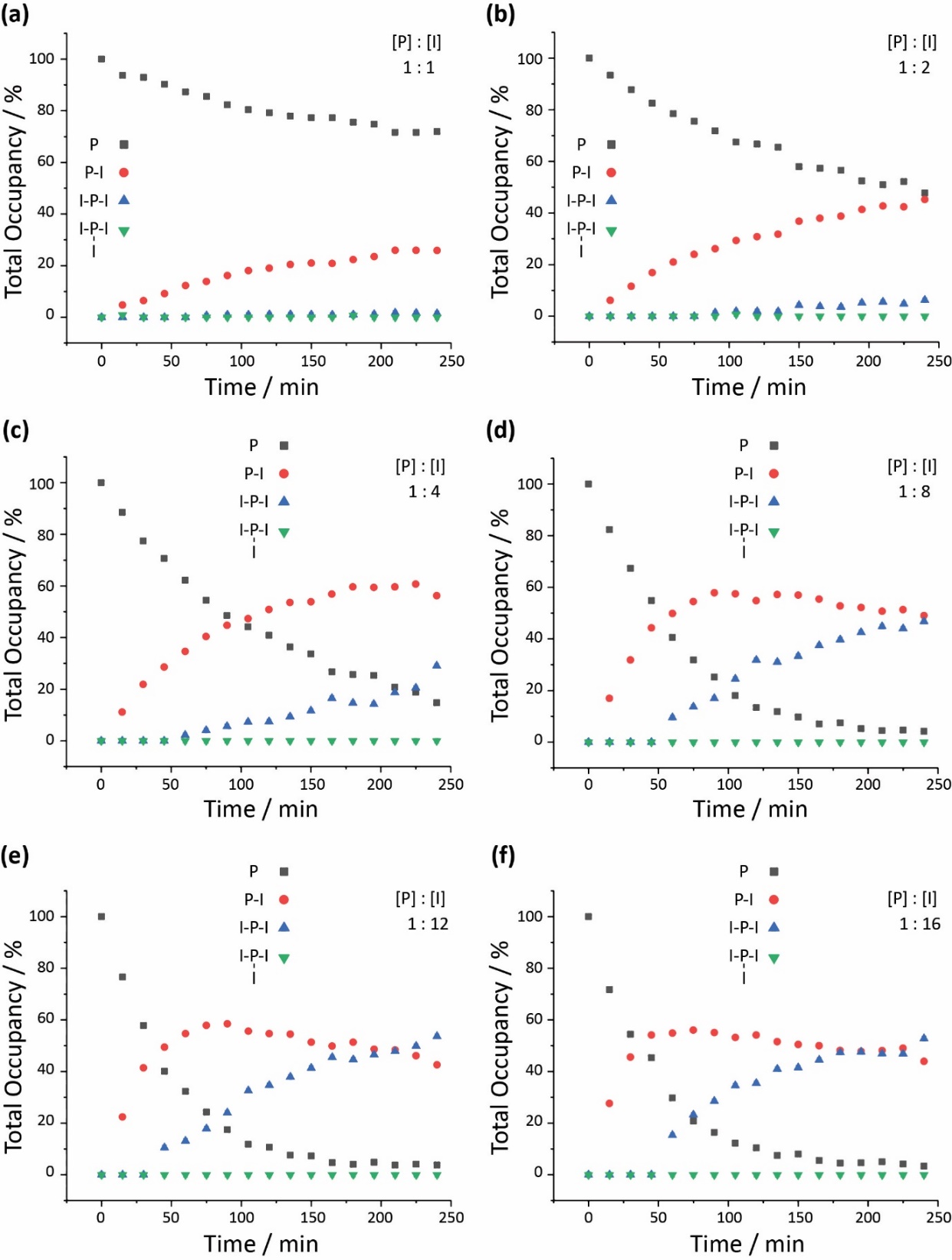


**Figure S9**. Plots of the % occupancy of the different protein states (P, P-I, I-P-I, etc.) over time for Aurora A (10 μM) in the presence of N-Myc_74-89 ASF_ at protein:inhibitor stoichiometries of (a) 1:1, (b) 1:2, (c) 1:4, (d) 1:8, (e) 1:12 and (f) 1:16 (25 mM Tris, 150 mM NaCl, 5 mM MgCl_2_, pH 7.5).

# **Attempted Site Mapping of Covalent Modification**

We explored a range of methods to try and identify the site of labelling. These included trypsin, chymotrypsin and proteinase K digests. Whilst we obtained good sequence coverage for Aurora-A in the absence of a modificaiton, we could not identify modified Aurora-A peptides from MS2 specta using automated data analyes.


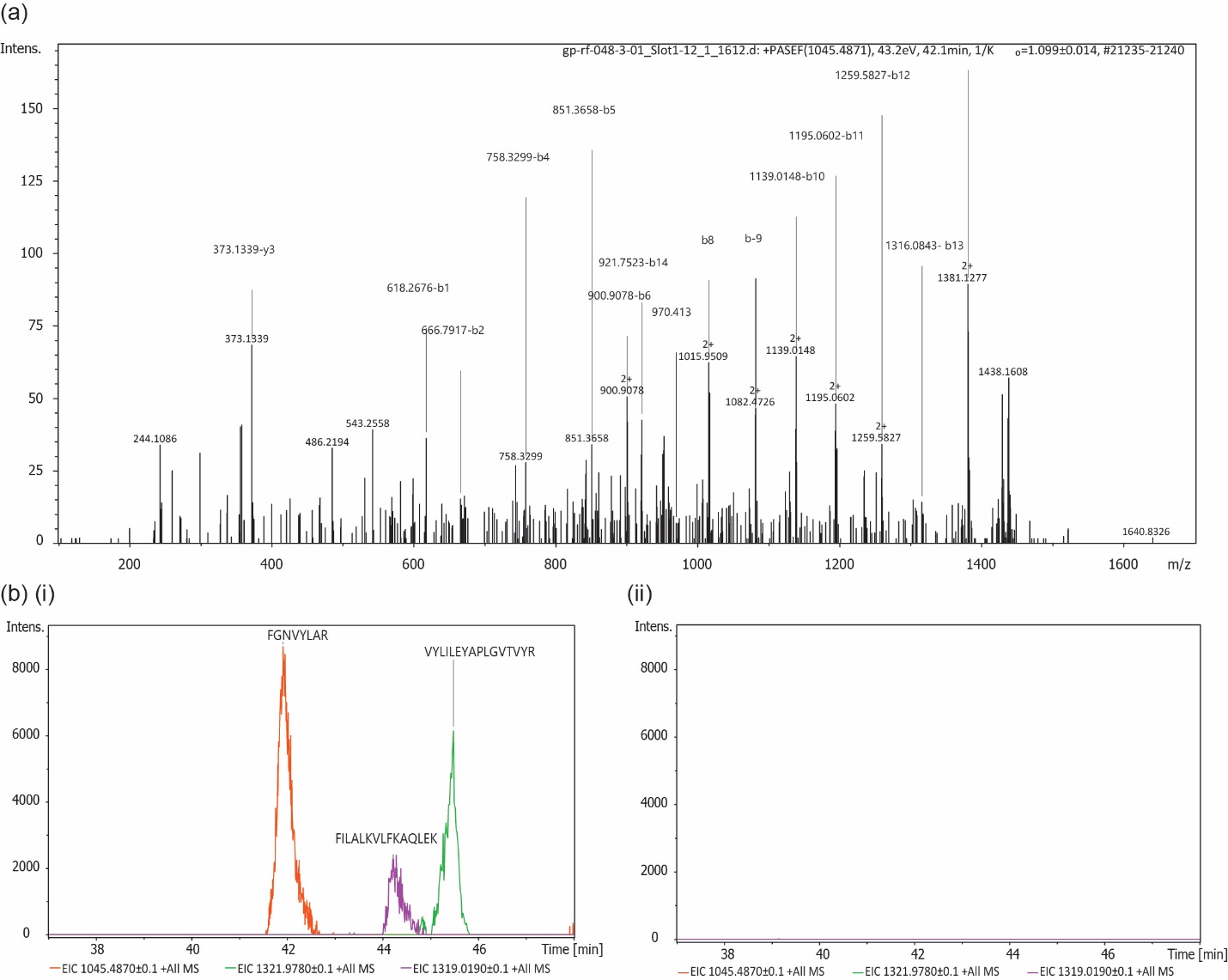


**Figure S10**. (a) Representative MS2 spectra for the fragmentation of the Myc_73-89 ASF_ labelled _no_FGNVYLAR_no_ fragment; (b) LC chromatograms corresponding to the ions from MS1 spectra corresponding to Aurora-A peptides in the (i) Myc_73-89 ASF_ labelled and (ii) Myc_73-89 ASF_ unlabelled samples

We were able to identify modified Aurora-A peptides by manual analysis of MS2 spectra of MS1 ions, however these were low abundance and so low confidence for a definitive or dominant site of covalent modificiation. The most promiment ion derives from modification of _144_FGNVYLAR_151_ by Myc_73-89 ASF_ for which annotated MS2 spectra are illustrated below. We also identified a modification of a different Tyr in _206_VYLILEYAPLGTVYR_220_, however the annotations were harder to identify manually in a noisier spectrum. Similarly we identified a modification on _157_FILALKVLFKAQLEK_171_ but these annotations were also harder to identify manually in a noisier spectrum. Upon comparison of the chromatograms for these compared to the control there are clear peaks in the MS1 that are not in the control sample.

# **Modified Protein Masses**

In the tables below, expected masses were calculated using the Lenntech molecular weight calculator (<https://www.lenntech.com/calculators/molecular/molecular-weight-calculator.htm>) based on chemical formulae obtained from the ExPASyProtParam tool (<https://web.expasy.org/cgi-bin/protparam/protparam>).

**Table S1 Protein HRMS Data**

|  |  |  |  | **Protein + N-Myc _73-89_ ASF** |  |  | **Protein + 2 × N-Myc _73-89_ ASF** |  |  |
| --- | --- | --- | --- | --- | --- | --- | --- | --- | --- |
| **Protein** | **Formula** | **Observed mass (Da)** | **Expected mass (Da)** | **Formula** | **Observed mass (Da)** | **Expected mass (Da)** | **Formula** | **Observed mass (Da)** | **Expected mass (Da)** |
| Aurora A _119-403C290A/C393A_ | C_1480_H_2325_N_413_O_421_S_6_ | 32832.0743 | 32832.11 | C_1480_H_2325_N_413_O_421_S_6_(C_100_H_139_N_21_O_31_S_2_) | 35027.6098 | 35027.53 | C_1480_H_2325_N_413_O_421_S_6_(C_100_H_139_N_21_O_31_S_2_)_2_ | 37223.1249 | 37222.95 |
| ADM22 | C_505_H_762_N_126_O_141_S_3_ | 10950.1175 | 10950.38 | C_505_H_762_N_126_O_141_S_3_(C_100_H_139_N_21_O_31_S_2_) | 13145.1510 | 13145.80 | C_505_H_762_N_126_O_141_S_3_(C_100_H_139_N_21_O_31_S_2_)_2_ | - | 15341.22 |
| *h*DM2_17-125_ | C_566_H_904_N_148_O_173_S_4_ | 12679.5341 | 12678.35 | C_566_H_904_N_148_O_173_S_4_(C_100_H_139_N_21_O_31_S_2_) | 14875.5097 | 14873.77 | C_566_H_904_N_148_O_173_S_4_(C_100_H_139_N_21_O_31_S_2_)_2_ | - | 17069.19 |
| MCL 1 | C_784_H_1257_N_227_O_236_S_4_ | 17768.3333 | 17766.96 | C_784_H_1257_N_227_O_236_S_4_(C_100_H_139_N_21_O_31_S_2_) | 19964.3333 | 19962.38 | C_784_H_1257_N_227_O_236_S_4_(C_100_H139N_21_O_31_S_2_)_2_ | - | 22157.80 |
| BCL-x_L_ affimer | C_609_H_926_N_166_O_172_S_3_ | 13420.9333 | 13421.04 | C_609_H_926_N_166_O_172_S_3_(C_100_H_139_N_21_O_31_S_2_) | 15616.8000 | 15616.46 | C_609_H_926_N_166_O_172_S_3_(C_100_H_139_N_21_O_31_S_2_)_2_ | - | 17811.88 |
| GFP-HIF1 ^L792A^ fusion construct | C_1630_H_2512_N_454_O_504_S_10_ | 36851.6417 | 36852.68 | C_1630_H_2512_N_454_O_504_S_10_(C_100_H_139_N_21_O_31_S_2_) | 39046.3021 | 39048.10 | C_1630_H_2512_N_454_O_504_S_10_(C_100_H_139_N_21_O_31_S_2_)_2_ | - | 41243.52 |

**Sequences**

**Aurora A _119-403_^C290A/C393A^**

GAMESKKRQWALEDFEIGRPLGKGKFGNVYLAREKQSKFILALKVLFKAQLEKAGVEHQLRREVEIQSHLRHPNILRLYGYFHDATRVYLILEYAPLGTVYRELQKLSKFDEQRTATYITELANALSYCHSKRVIHRDIKPENLLLGSAGELKIADFGWSVHAPSSRRTTLAGTLDYLPPEMIEGRMHDEKVDLWSLGVLCYEFLVGKPPFEANTYQETYKRISRVEFTFPDFVTEGARDLISRLLKHNPSQRPMLREVLEHPWITANSSKPSNAQNKESASKQS

**ADM22**

SENSLEIEELARFAVDEHNKKENALLEFVRVVKAKEQMGVNPEEMQTMYYLTLEAKDGGKKKLYEAKVWVKWWWGFHIWDNFKELQEFKPV

**hDM2 *^res^* ^17-125^**

GTSQIPASEQETLVRPKPLLLKLLKSVGAQKDTYTMKEVLFYLGQYIMTKRLYDEKQQHIVYCSNDLLGDLFGVPSFSVKEHRKIYTMIYRNLVVVNQQESSDSGTSVSEN

**MCL 1**

SELYRQSLEIISRYLREQATGAKDTKPMGRSGATSRKALETLRRVGDGVQRNHETAFEGMLRKLDIKNEDDVKSLSRVMIHVFSDGVTNWGRIVTLISFGAFVAKHLKTINQESCIEPLAESITDVLVRTKRDWLVKERGWDGFVEFFHVEDLEAA

**BCL-xL affimer**

MASAATGVRAVPGNENSLEIEELARFAVDEHNKKENALLEFVRVVKAKEQMFSWLDWEETMYYLTLEAKDGGKKKLYEAKVWVKPALLWSPHGNFKELQEFKPVGDAAAAAAHHHHHH

**GFP-HIF1 ^L792A^ fusion construct**

MGHHHHHHHHHSSGHENLYFQGTVSKGEELFTGVVPILVELDGDVNGHKFSVSGEGEGDATYGKLTLKFICTTGKLPVPWPTLVTTLTYGVQCFSRYPDHMKQHDFFKSAMPEGYVQERTIFFKDDGNYKTRAEVKFEGDTLVNRIELKGIDFKEDGNILGHKLEYNYNSHNVYIMADKQKNGIKVNFKIRHNIEDGSVQLADHYQQNTPIGDGPVLLPDNHYLSTQSALSKDPNEKRDHMVLLEFVTAAGITLGMDELYKGGGTNNLGILEVLFQGPGSSDLACRLLGQSRDESGAPQLTSYDCEVNAGIQGSRNLLQGEELLRALDQVN

# **Peptide Characterization Data**

Peptide purity was confirmed by analytical HPLC. Here, HRMS data and analytical HPLC spectra of the peptides are provided.

Below are tabulated HRMS data for peptidomimetics. Peptide identity was confirmed by the inspection of multiple charge states which are quoted as the monoisotopic peak for the Expected (Exp^d^) and Observed (Obs^d^) masses. HRMS and analytical HPLC spectra are provided in the Appendix.

**Table S2**

|  | **[M+H]^1+^ Obsd** | **[M+H]^1+^ Exp** | **[M+H]^2+^ Obsd** | **[M+H]^2+^ Exp** | **[M+H]^3+^ Obsd** | **[M+H]^3+^ Exp** |
| --- | --- | --- | --- | --- | --- | --- |
| **N-Myc_73-89_** | 2070.9990 | 2070.0841 | 1036.4995 | 1036.4921 | 691.3342 | 691/3280 |
| **N-Myc_73-89 ASF_** | 2214.9652 | 2214.9522 | 1108.4826 | 1108.4761 | 739.3210 | 739.3174 |
| **N-Myc_74-89 ASF_** | 2085.9240 | 2085.9096 | 1043.9620 | 1043.9548 | 696.3073 | 696.3032 |

**N-Myc _61-89_ FAM**


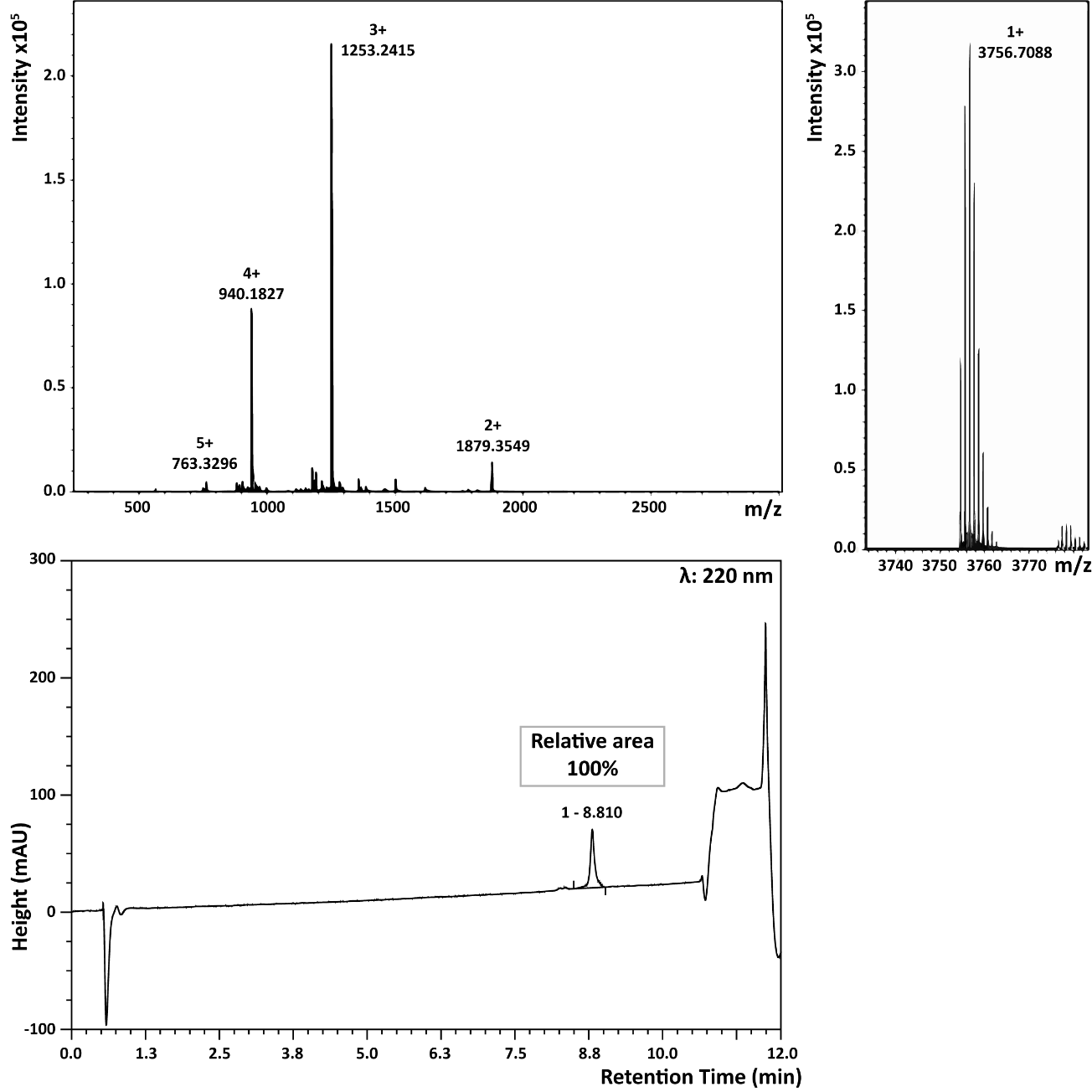


**N-Myc _73-89_**


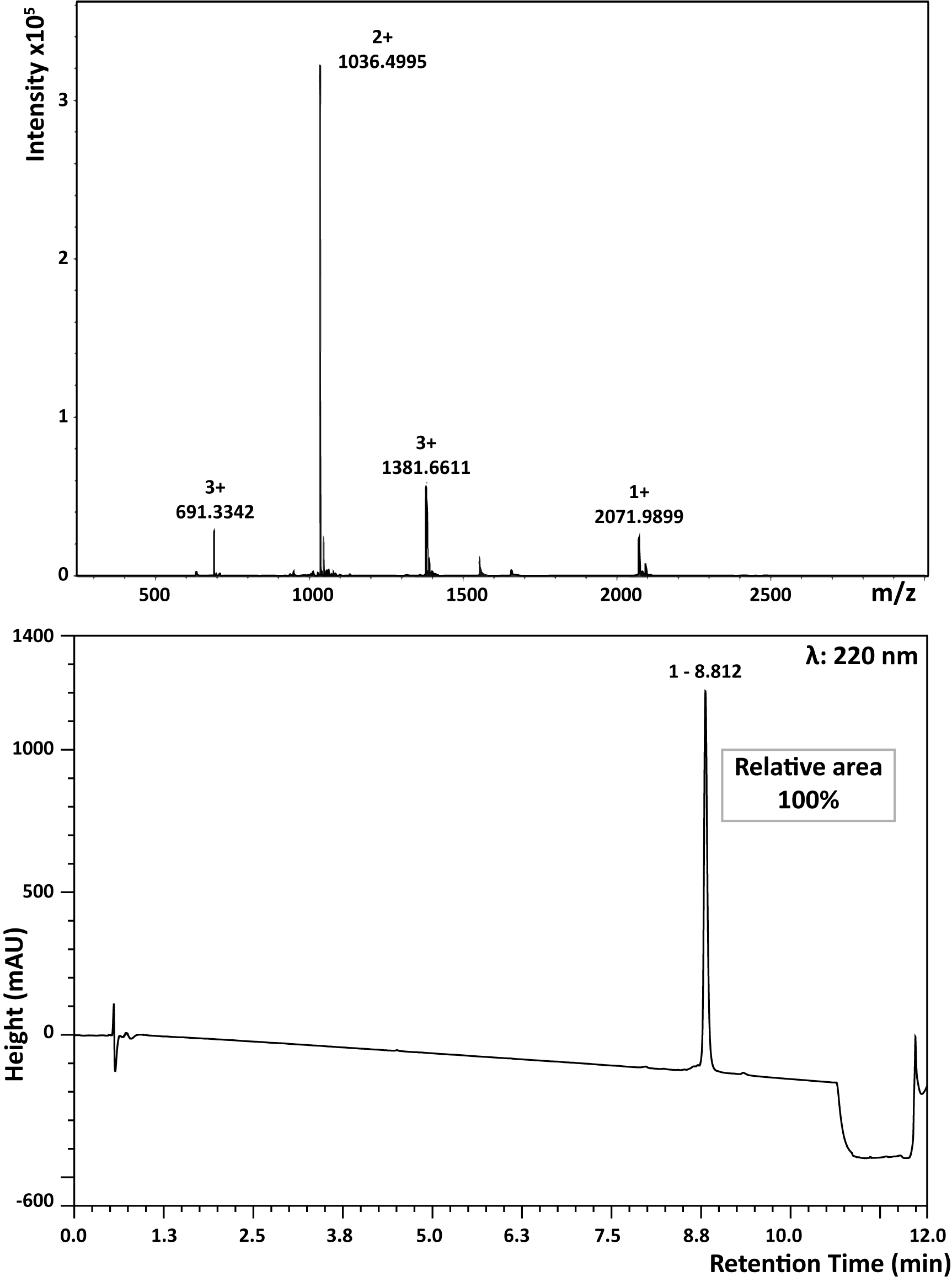


**N-Myc _73-89_ ASF**


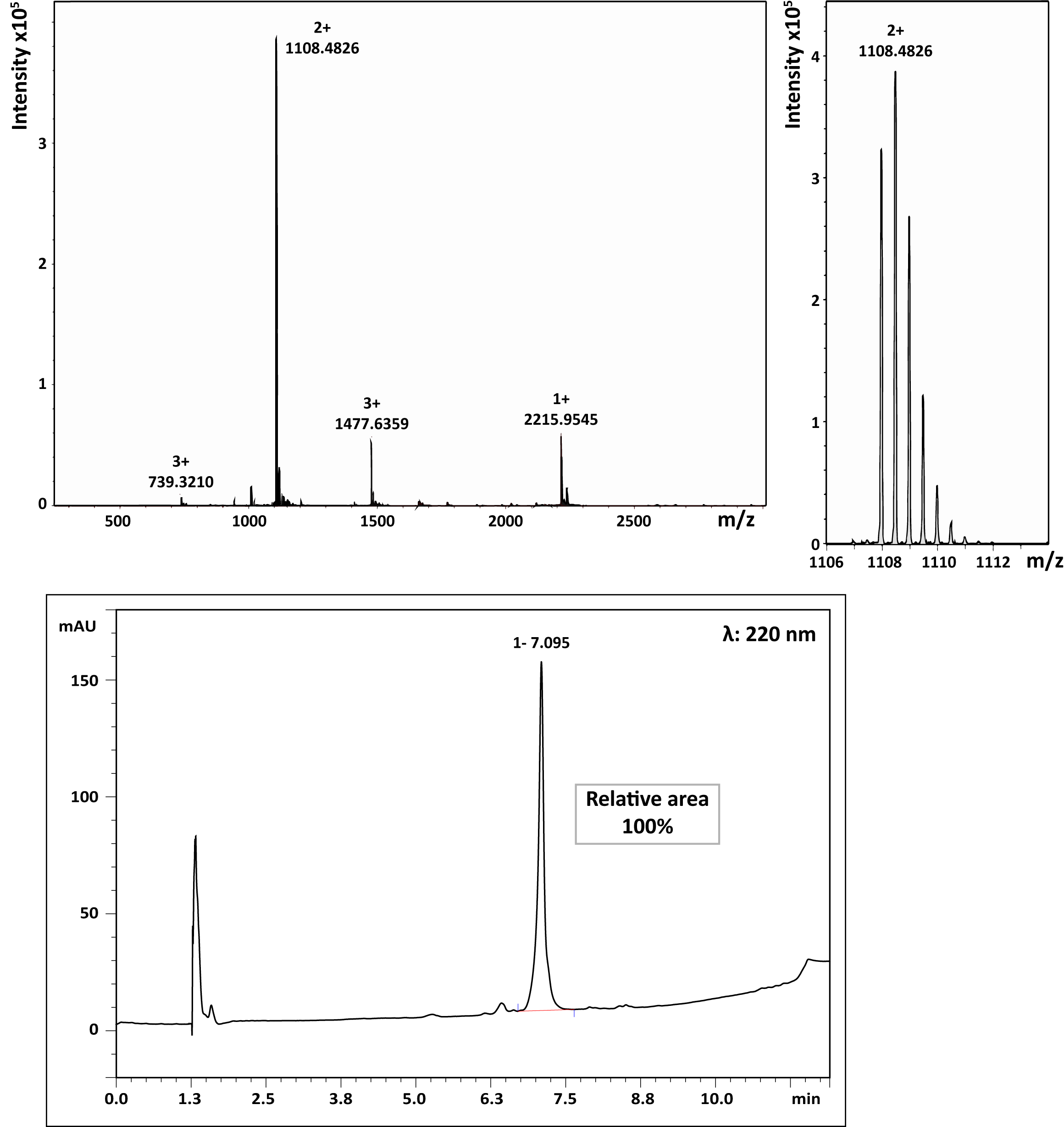


**N-Myc _74-89 ASF_**


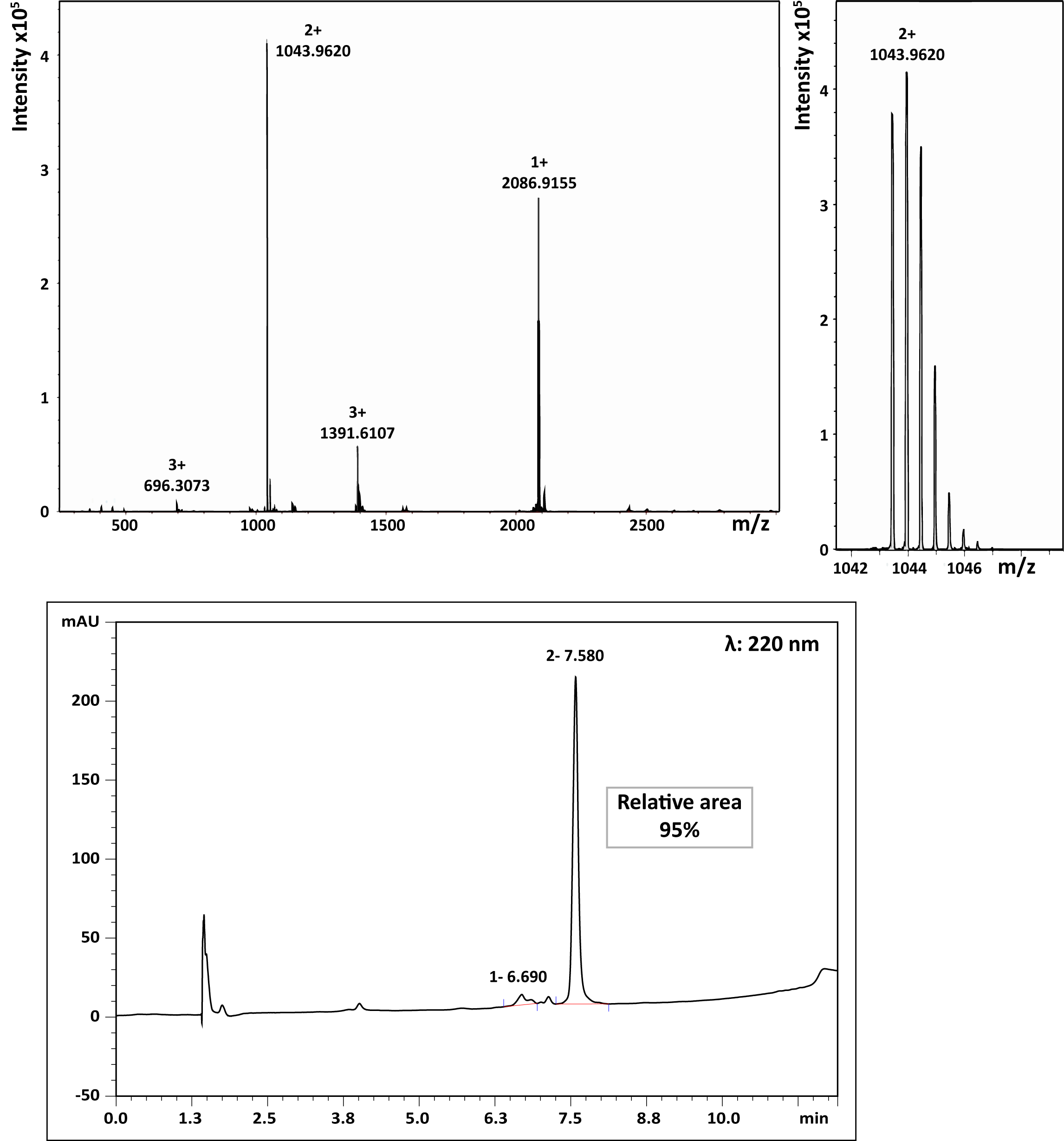

Supplement: Supplementary file 1 — Figure S1: Mass spectrometry analyses of N‐Myc73–89 ASF in the presence of Aurora‐A at 0, 1 and 3 h ([peptide] = 45 μM] [protein] = 90 μM, 25‐mM Tris, 150‐mM NaCl, 5‐mM MgCl2 and pH 7.5). Figure S2: Mass spectrometry analyses of N‐Myc73–89 ASF in the presence of ADM22 at 0, 1 and 3 h ([peptide] = 45 μM] [protein] = 90 μM, 25‐mM Tris, 150‐mM NaCl, 5‐mM MgCl2 and pH 7.5). Figure S3: Mass spectrometry analyses of N‐Myc73–89 ASF in the presence of hDM2 at 0, 1 and 3 h ([peptide] = 45 μM] [protein] = 90 μM, 25‐mM Tris, 150‐mM NaCl, 5‐mM MgCl2 and pH 7.5). Figure S4: Mass spectrometry analyses of N‐Myc73–89 ASF in the presence of BCL‐xL at 0, 1 and 3 h ([peptide] = 45 μM] [protein] = 90 μM, 25‐mM Tris, 150‐mM NaCl, 5‐mM MgCl2 and pH 7.5). Figure S5: Mass spectrometry analyses of N‐Myc73–89 ASF in the presence of GFP‐HIF‐1αL792A at 0, 1 and 3 h ([peptide] = 45 μM] [protein] = 90 μM, 25‐mM Tris, 150‐mM NaCl, 5‐mM MgCl2 and pH 7.5). Figure S6: Mass spectrometry analyses of N‐Myc73–89 ASF in the presence of MCL‐1 at 0, 1 and 3 h ([peptide] = 45 μM] [protein] = 90 μM, 25‐mM Tris, 150‐mM NaCl, 5‐mM MgCl2 and pH 7.5). Figure S7: Mass spectrometry analyses of Aurora‐A (10 μM) incubated with (a) 20‐μM N‐Myc73–89 ASF and (b) 20‐μM N‐Myc74–89 ASF after 2 h (25‐mM Tris, 150‐mM NaCl, 5‐mM MgCl2 and pH 7.5). Figure S8: Plots of the % occupancy of the different protein states (P, P‐I, I‐P‐I, etc.) over time for Aurora‐A (10 μM) in the presence of N‐Myc73–89 ASF at protein:inhibitor stoichiometries of (a) 1:1, (b) 1:2, (c) 1:4, (d) 1:8, (e) 1:12 and (f) 1:16 (25‐mM Tris, 150‐mM NaCl, 5‐mM MgCl2 and pH 7.5). Figure S9: Plots of the % occupancy of the different protein states (P, P‐I, I‐P‐I, etc.) over time for Aurora‐A (10 μM) in the presence of N‐Myc74–89 ASF at protein:inhibitor stoichiometries of (a) 1:1, (b) 1:2, (c) 1:4, (d) 1:8, (e) 1:12 and (f) 1:16 (25‐mM Tris, 150‐mM NaCl, 5‐mM MgCl2 and pH 7.5). Figure S10: (a) Representative MS2 spectra for the fragmentation of the Myc73–89 ASF [file PSC-32-e70086-s001.docx]
